# Machine learning enabled orthogonal camera goniometry for accurate and robust contact angle measurements

**DOI:** 10.1038/s41598-023-28763-1

**Published:** 2023-01-27

**Authors:** Hossein Kabir, Nishant Garg

**Affiliations:** Department of Civil and Environmental Engineering, University of Illinois at Urbana-Champaign, Urbana, IL USA

**Keywords:** Civil engineering, Characterization and analytical techniques, Imaging techniques, Computational science, Imaging and sensing

## Abstract

Characterization of surface wettability plays an integral role in physical, chemical, and biological processes. However, the conventional fitting algorithms are not suitable for accurate estimation of wetting properties, especially on *hydrophilic* surfaces, due to optical distortions triggered by changes in the focal length of the moving drops. Therefore, here we present an original setup coupled with Convolutional Neural Networks (CNN) for estimation of Contact Angle (CA). The developed algorithm is trained on 3375 ground truth images (at different front-lit illuminations), less sensitive to the edges of the drops, and retains its stability for images that are synthetically blurred with higher Gaussian Blurring (GB) values (GB: 0–22) if compared to existing goniometers (GB: 0–12). Besides, the proposed technique can precisely analyze drops of various colors and chemistries on different surfaces. Finally, our automated orthogonal camera goniometer has a significantly lower average standard deviation (6.7° vs. 14.6°) and coefficient of variation (14.9 vs. 29.2%) than the existing techniques and enables wettability assessment of non-spherical drops on heterogeneous surfaces.

## Introduction

Wettability can be defined as the preference of a liquid to hold contact with the surface of a solid, governed by not only the minimization of surface free energy in a ternary solid–liquid-gas system but also by the history of the system. This phenomenon, measured by contact angle (CA), i.e., the angle between the liquid–vapor interface and solid–liquid interface, has been studied over the past two centuries^1–5^. Broadly, wettability depends on the combination of materials (solid and liquid) that impacts a range of diverse phenomena ranging from disruption of gastric mucosa hydrophobicity via aspirin^6^ to cell-biomaterial interactions^7^, as well as, efficiency of graphene-based microfluidics^8^ to sintering and designing of metallic nanoparticles^9^ and polymeric surfaces^10, 11^. Specifically, to design and fabricate a variety of modern materials with engineered wettability, such as, superhydrophobic microfluidic devices^12^, efficient fog harvesting materials^13^, novel phase change heat transfer systems^14^, and self-cleaning coatings^15^, surface sciences play a central role^16^. Surface wettability is controlled by both the *adhesive* forces between the solid and the liquid, which promote drop spreading, and the *cohesive* forces within the liquid, which limit the spreading^17^. Characterization of surface wettability is commonly achieved by the use of CA measurements which are found to be correlated with the surface tension. Neglecting gravity, the CA of a liquid drop placed on a solid surface under mechanical equilibrium can pinpoint the mean solid–liquid interfacial energy and is explicitly formulated in Young's equation^1, 18, 19^:

1 $$\mathrm{cos}{\theta }_{y}= \frac{{{\gamma }_{sg}- \gamma }_{sl}}{{ \gamma }_{lg}}$$

where, ${\theta }_{y}$ is Young's CA made by the drop on the solid surface at the ternary phase contact line. In addition, ${\gamma }_{sg}$, ${\gamma }_{sl}$, ${\gamma }_{lg}$ represent solid–gas, solid–liquid, and liquid–gas interfacial tensions, respectively. It should be noted that (in experiments) attaining a global equilibrium CA configuration on ideal solid surfaces is almost unachievable. In fact, any kinetically stable drop configuration between the receding and advancing configurations must be thermodynamically considered a metastable equilibrium configuration as it is not possible to measure the energy of solid–liquid systems at this state^20–22^. Hydrophobic or nonwetting surfaces have CAs larger than 90°, while CAs less than 90° correspond to hydrophilic surfaces^23^. Among most of the existing instruments such as dynamic sessile drop, dynamic Wilhelmy, single-fiber Wilhelmy, and single-fiber meniscus methods, the "sessile drop" techniques coupled with optical image analysis methods have been widely accepted and used due to their simplicity for direct measurements of tangent angles^24–28^.

In the sessile drop method, various fitting algorithms are commonly used with a broader utility to determine the CA of a drop profile^29^. Among these algorithms, circular-fitting^24^, ellipse-fitting^30^, axisymmetric drop shape analysis (ADSA)^31^, and polynomial-fitting algorithms^32^ are widely used to numerically generate the drop profiles. Specifically, the circular-fitting method assumes that the profile is part of a circle and leverages the Levenberg–Marquardt optimization to obtain the circular equation for estimating the CA. However, the method can only provide accurate wettability estimations of drops with relatively small CAs, e.g., < 60°. Furthermore, the ellipse-fitting algorithm uses implicit second-order polynomial for accurate analysis of drops with relatively larger CA, e.g., > 90°^33^. In the ADSA method, the Laplace equation is numerically solved to precisely capture a more complex axisymmetric drop profile, though it has two main limitations: (1) the drop’s apex must be fully visible, and (2) only axisymmetric drops are resolvable. Finally, the polynomial fitting method obviates the need for estimating the drop profile equation and is commonly used due to its simplicity. However, this method is highly sensitive to the order of polynomial and the number of pixel points. For this reason, it is recommended to improve the accuracy of measurements by implementing subpixel resolution and subsequently extrapolating the coordinates of the contact points^34, 35^. But even using a high subpixel resolution technique for detecting the edges, the accuracy will be still at the pixel level since the ill-defined edges can potentially disturb the shape of the drop. Furthermore, the subpixel edge detection method can be done in various ways, leading to inaccurate and subjective estimates^36–38^. In summary, none of these introduced fitting techniques are capable of detecting almost flat drops, i.e., CA < 20°, due to the high uncertainty associated with the tangent line allocation. And the dependency of all these methods on the drop profile can cause systematic errors, especially in the presence of optical noises (e.g., diffracting, scattering, and blurring)^39, 40^. In particular, the rapid movement of drops on porous hydrophilic surfaces would blur the imagery data due to the changes in the focal length of the camera, and the traditional fitting algorithms cannot be used to accurately delineate the region of interest (ROI). Moreover, the existing fitting algorithms cannot be used to determine the parameters of dynamics contact angle, i.e., advancing and receding angles, especially if the syringe remains in the drop or if the drop is not fully visible by the camera. Therefore, there is a need to develop a robust method that is based on multi-layer neural networks to recognize visual patterns (with minimal preprocessing) and to automatically detect and analyze drops of varying wettability for static and dynamic CA measurements, being independent of the skills and experience of the operator and is less sensitive to the variations in the camera focus and lighting conditions. In other words, if the CNN model becomes fully trained, the model can provide more accurate measurements than experienced users, hence it can exceed human-level performance.

As a result, this study presents a simple, yet robust setup coupled with the Convolutional Neural Networks (CNN) for direct and accurate wettability assessment on both hydrophobic (θ > 90°) and hydrophilic (θ < 90°) surfaces. Specifically, we have developed a simple and original setup, which consists of two USB microscope cameras with front-lit LEDs ($25 each) and a Z-axis manual mechanical stage ($125), which in total costs less than $200. The proposed setup can provide two independent views of the drops, which can potentially minimize intrinsic optical errors and maximize the performance of measurements on heterogeneous surfaces. Also, the developed CNN model is capable of fast bulk processing of the image data in real-time. As it is not strictly necessary to fine-tune the hyperparameters of our model during the training process, instead of using a computationally expensive k-fold cross-validation, a hold-out set is commonly used to randomly split up the dataset into a 'train' and 'test' set. Since our dataset contains almost nine similar scenarios for every drop image (to account for various lighting conditions), it would be impossible to select an unbiased subset of the test set, which has not been used at all in the train set. Therefore, to avoid getting a false impression of better accuracy, all the 3375 ground truth images are assigned to the train set. Also, for generalization, a completely new test set with 70 binarized drop images is used to determine whether the predicted tangent lines match the ImageJ estimations. To benchmark our methodology, the results of the present analysis are compared with that of a commercially available goniometer fabricated by Ramé-hart Instrument Co., which analyzes the drops using a built-in DROPimage Advanced (DROPiA) software, i.e., based on the fitting algorithm.

A direct comparison between our proposed and existing commercial systems reveals three major advantages of our system. Firstly, on one hand, for *hydrophobic* surfaces (θ > 90°), in terms of accuracy, both the existing and proposed systems have a similar average standard deviation (9.4° vs. 9.7°) and coefficient of variation (8.9 vs. 9.0%). However, on the other hand, for *hydrophilic* surfaces (θ < 90°), our proposed system has a significantly lower average standard deviation (6.7° vs. 14.6°) and coefficient of variation (14.9 vs. 29.2%) compared to the existing system. This significantly improved accuracy of our proposed system is highly beneficial for hydrophilic samples. Secondly, our proposed goniometer is also highly robust as it is capable of accurate CA measurements [in a broader range of Gaussian Blurring (GB) index values] of a convoluted image. Specifically, while the existing commercial system fails to fit a tangent line beyond a GB value of 12 (working range is 0–12), our new proposed system works effectively up to a GB value of 22 (working range is 0–22), proving its robustness. Thirdly and finally, our proposed setup can determine surface heterogeneity of solids by analyzing non-spherical drops via orthogonal viewpoints which is also a considerable advance over a single view goniometer. In summary, these results introduce a powerful CNN-enabled goniometer for real-time characterization of the wettability phenomena on solids of varying wettability.

## Results

### Impact of room lighting condition

**Figure 1:**
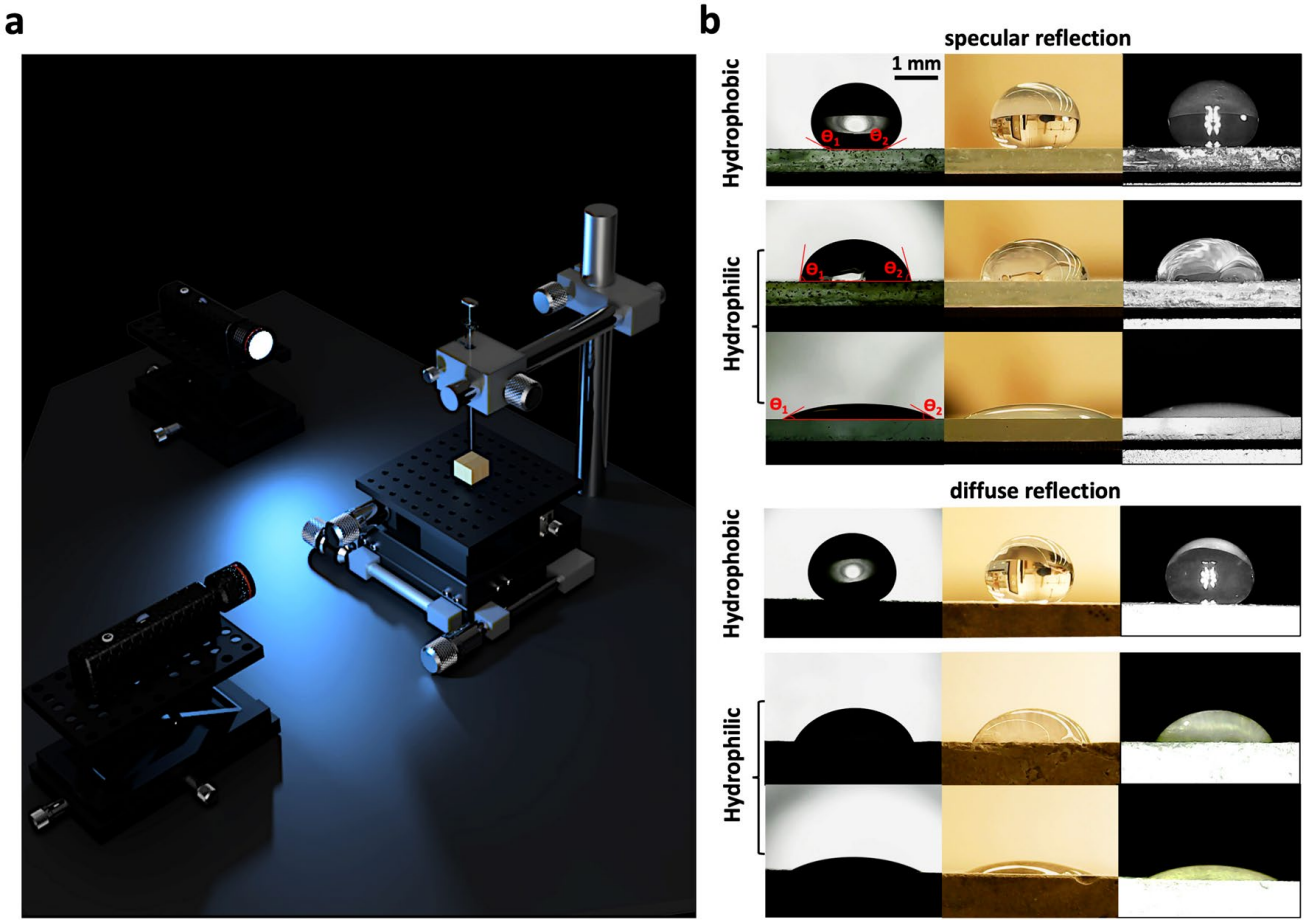
CA measurements via sessile drop method using an orthogonal camera setup. (**a**) A 3D schematic of the proposed setup that comprises two front-lit USB microscope cameras and a single XYZ mechanical stage placed in a dark room, (**b**) estimating CA on hydrophobic (top row) and hydrophilic (second and third rows) surfaces through back-lit (left column), normal lighting (middle column), and front-lit (right column) setups for drops sitting on substrates with specular (i.e., nonporous ceramic) or diffuse (i.e., porous ceramic) reflections.

Wettability measurements would be both accurate and reproducible if the location of the light source and the lighting condition of the surroundings are properly chosen^41, 42^. Analysis of back-lit drops is a widely accepted practice for better distinguishing the boundaries of a drop profile^43, 44^ (Fig. 1b, left columns: surfaces with specular or diffuse reflections). However, coupling a secondary camera system to the back-lit drop is not achievable as it can change the apparent boundary of drops. In other words, back-lit illumination can only work if a drop is analyzed in a single direction (Fig. S1a); however, false boundaries would appear on the edges of the drop if another light source is added perpendicularly to the original direction (Fig. S1b). Figure S1c,d also explain the mechanisms of backlit illumination for single and double light sources and justify how the illumination rays can be potentially reflected from the drop surface and detected by the adjacent camera. In fact, the dual-camera system for analysis of the back-lit drop has been reported to be effective only when CA measurements are not simultaneously taken from various angles^45^. Therefore, the proposed setup provides an effective solution for simultaneous measurements of CAs at various angles (Fig. 1a).

**Figure 2:**
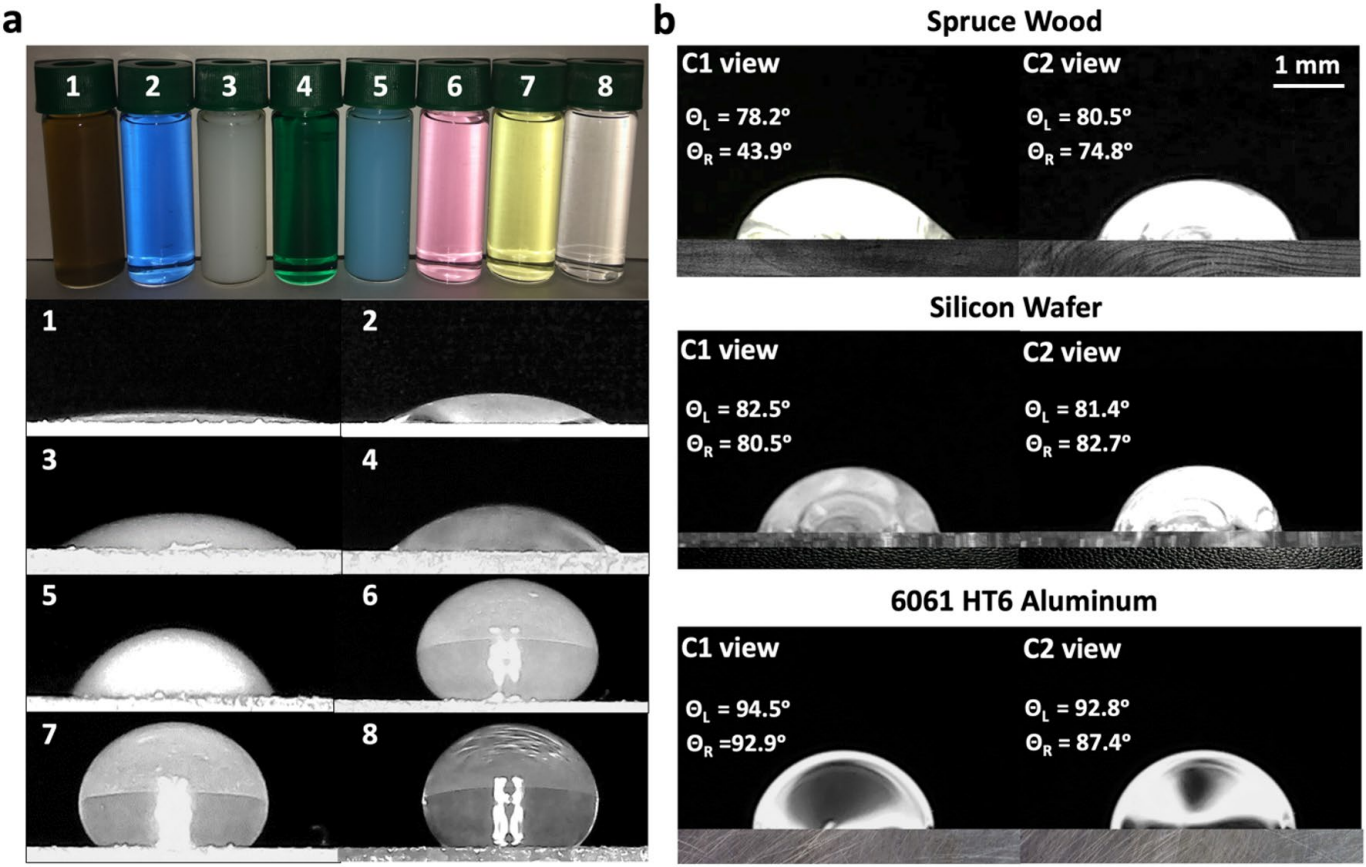
Applicability of front-lit illuminations for CA measurements: (**a**) liquids of varying compositions and colors, where specifically 1 is WD-40 lubricant, 2 is alcohol, 3 is a commercial siloxane, 4 and 5 are polishing oils, 6 and 7 are buffer solutions with pH of 4.0 and 7.0, respectively, and 8 is deionized water on glass slides, (**b**) CA measurements on porous and nonporous surfaces, i.e., spruce wood, silicon wafer, and 6061 HT6 aluminum, showing a clear recognition of the front-lit drops.

Followed by ensuring the proper location of the illumination source, it is equally important to specify the correct room lighting condition that surrounds the drop. Figure 1b (middle columns) shows CA measurements on substrates with either specular or diffuse reflections in a normal lighting room condition. In this condition, however, the light transition between the drop and the background is not sharp enough to delineate the boundaries of the drops. Consequently, the front-lit technique (in a dark room) is used in this study for all CA measurements as it provides clearer boundaries (see Fig. 1b, right columns). Additionally, Fig. 2b proves the applicability of the developed front-lit setup for wettability assessment of different porous or nonporous substrates, e.g., spruce wood, silicon wafer, and 6061 HT6 aluminum alloy surfaces, due to the distinguishable boundary of the drops. As shown in Fig. 2b (first row, i.e., Spruce Wood), there is a significant difference between the CA values taken from C1 (camera 1) and C2 (camera 2) views. Subsequently, at least two *orthogonally* aligned cameras should be used to capture the surface heterogeneity by covering a significantly large portion of the drop. It is worth noting that in this dual-camera system, the presence of a third camera would be redundant and does not provide additional measurements. Moreover, the proposed technique is also proven to be applicable to all liquids regardless of their chemistries and colors, see Fig. 2a.

### Automated CA measurements using CNN

**Figure 3:**
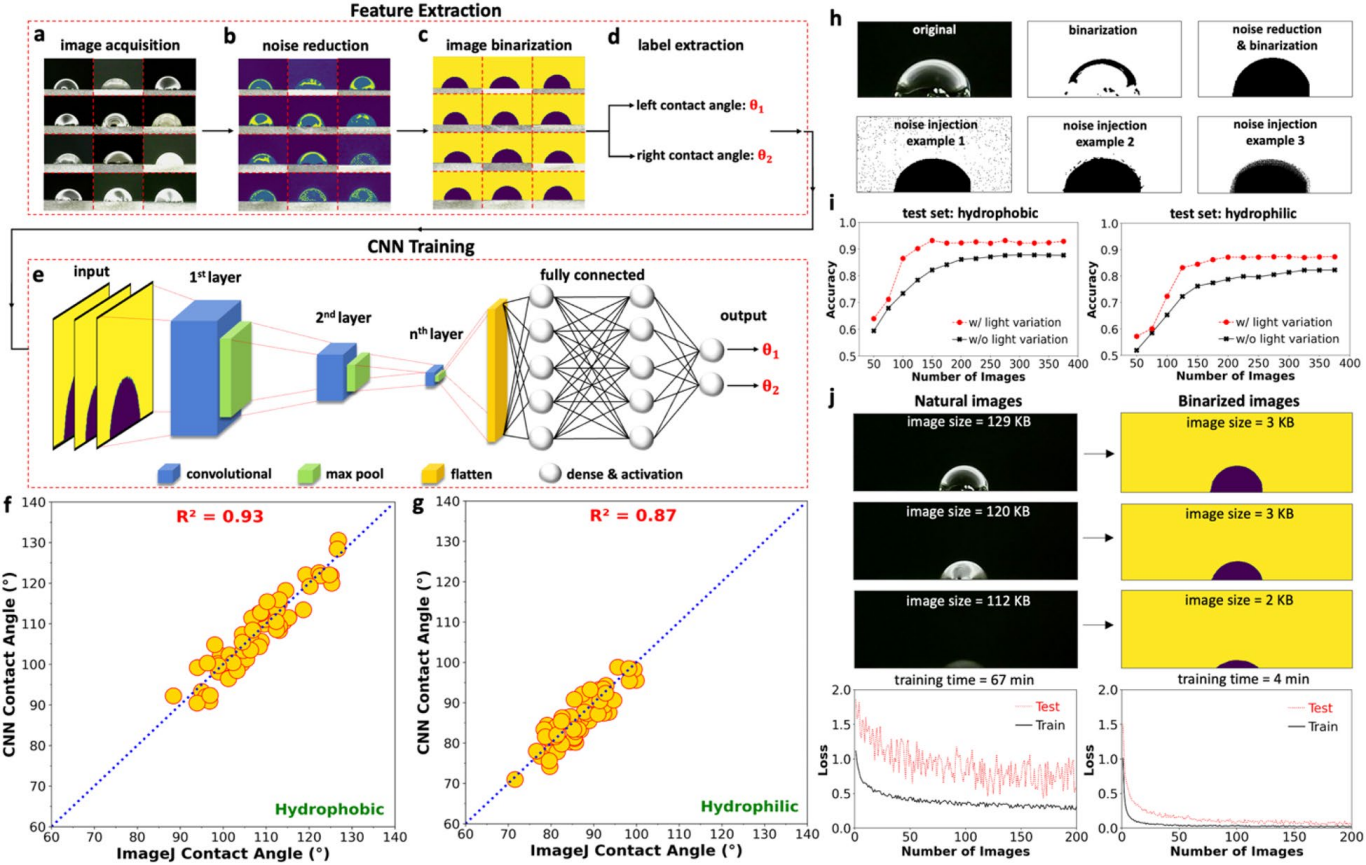
Workflow for feature extraction and training the CNN model: (**a**) acquiring natural front-lit drop images in a dark room, (**b**) denoising the images using a filter in Python, (**c**) converting the multi-tone to bi-tonal (binary) images to better distinguish the overall geometry of drops, (**d**) estimating the left and right CAs of each drop using the Point Picker plugin in *ImageJ* to create the ground truth dataset, and (**e**) designing an nth layer CNN framework to estimate the left and right CAs, (**f**) matching the CNN with the *ImageJ* CA estimations on hydrophobic, and (**g**) hydrophilic surfaces. (**h**) Top row: noise reduction filter on natural images to create ground truth binary images prior to the training step, bottom row: three different examples of noise injection filter on binary images during the data augmentation step, (**i**) accuracy of the CNN model as a function of the number of unique training images (solid black line) for hydrophobic (left subplot) and hydrophilic (right subplot) test sets, in which the 9 variations in the lighting conditions (red dashed line) has proven to improve the accuracy and stability of results, and (**j**) estimating the performance of the CNN model on natural (left column) or binarized (right column) images proving that binarizing the input data would reduce the training time and improve the accuracy/stability of the CA estimations.

The repeatability and reproducibility of the CAs estimated by commercially available goniometers can be largely influenced by the skills and experience of the operator and hence can be subjective. Specifically, the way an operator aligns the baselines and focuses the camera on the ROI controls the overall accuracy of the measured tangent lines at the solid–liquid interface^46, 47^. As a result, followed by specifying the proper illumination conditions, it is now necessary to develop a fully automated image-based technique that can analyze the drops from image data. For this purpose, this section details a unique procedure (using feature extraction and training stages) that can be leveraged to autonomously measure the CA of drops on hydrophobic and hydrophilic surfaces. During the feature extraction stage, images of front-lit drops are imported (Fig. 3a), and their reflections are removed using a noise reduction filter imported from OpenCV library^48^, see Fig. 3b. This enhancement filter minimizes the artifacts from the raw images and maximizes the signal-to-noise (SNR) ratio, i.e., expressed in decibel (dB) and is defined as SNR = 20 log_10_ (A_signal_/A_noise_), where A is the root mean square amplitude of the signal. Especially, this step is necessary during the early stages (first 10 s) of solid–liquid interaction (Fig. S2).

As shown in Fig. 3c, the noise-reduced images are then binarized using a bi-color toning filter^49^ such that the overall geometry of each drop can be fully recognized. Then, using the "Point Picker" plugin in the *ImageJ* software^50^, the points along the edge of the drops are manually selected to establish the best-fit tangent line for CA estimation of asymmetric drops. It should be noted that the combination of noise reduction and binarization filters does not change the tangent angles at the ternary phase contact line, see Fig. S3. In the next step, 375 different binary images are selected, and prior to binarization, their brightness is changed from 80% darker to 80% brighter (i.e., 375 × 9 = 3375 images in total), see Fig. S4a. Specifically, as shown in Fig. S4c, the binarized map of each image at different lighting conditions does not perfectly match resulting in a small variation in the manually estimated left and right CAs (Fig. S4b). This would further generalize the model against the variation in the front-lit (LED) illumination conditions. As a result, in the last step of the feature extraction stage (Fig. 3d), 3375 images are manually measured and labeled as ground truth input data to train the CNN model. The experience of the person who is training the dataset plays a significant role in the accuracy of results. Although, in reality, it is not possible to create *pure objective* ground truth data, this set can be *good enough* to create a nonfatal and functional model that provides sufficiently accurate estimates for the test set. For this purpose, an *experienced* user used Image J to manually (or artificially) measure the binary images in order to create a highly accurate, unbiased, representative, and objective ground truth/ training set. As shown in Fig. S5b, if the ground truth data is prepared by inexperienced users (i.e., users 1 and 2), the results would be less reliable (with a larger standard deviation, see Fig. S5d) than those estimated by experienced users (i.e., users 3 and 4). In other words, the coefficient of variation, i.e., the ratio of standard deviation to mean, of the estimates provided by the inexperienced group would be ~ 3 times larger than that of the experienced group. However, as soon as the CNN algorithm becomes fully trained (Fig. S5a), the results would be even more accurate.

Moving on to the training stage, the developed CNN model comprises multiple layers of nonlinear transformation (Fig. 3e), which can iteratively learn how to do CA measurements using the overall geometry of the recognized drops from the binarized images. This transformation comprises five main components: fully connected layers, convolutional layers, activation functions, pooling layers, and regularization such that the predicted values would be close enough to the expected target^51^. Specifically, the fully connected layers connect every layer to each activation unit of the subsequent layer, while not preserving the spatial structure of the input value. In contrast, the convolutional layers act as a filter that preserves the spatial relationship between input and feature map and enables weight sharing to improve the learning convergence^52, 53^. It is worth noting that both the noise reduction (prior to model training) and noise injection (during data augmentation) filters are critical for maximizing the model accuracy. In particular, the noise reduction filter on natural images makes the boundary of binarized drop images clearer at the ternary phase contact line for accurate manual CA estimations, while noise injection can make the model robust against optical distortions see Fig. 3h. Followed by training the CNN model, Fig. 3f,g matches 70 CA estimations (test set) from the CNN and *ImageJ,* implying a relatively high R-squared of 0.93 and 0.87 for hydrophobic and hydrophilic surfaces, respectively. Besides, Video 1 shows an automated CNN-based CA measurement of a water drop on a porous hydrophilic surface using the developed setup, which proves the efficiency of the developed algorithm. As shown in Fig. S6 (which is a snapshot of Video 1), the maximum difference in CA estimated by the two methods, i.e., CNN and ImageJ, is never more than ~ 4° for symmetrical drops. Similarly, the high accuracy of the proposed method for the analysis of non-symmetrical drops is shown in Video 2 and Fig. S7 (which is a snapshot of Video 2). Basically, Fig. S7 shows that non-symmetrical back-lit drops rolling down a sloped stage (Fig. S7a) or vertical wall (Fig. S7b) were accurately analyzed. In fact, comparisons of Image J and CNN-based estimations show a small discrepancy of < 3° proving the robustness of the proposed model for analysis of both symmetrical and non-symmetrical drops. However, the differences between these two techniques (Image J and CNN) cannot be zero since the established R-squared values (between the CNN and ImageJ algorithms) are smaller than unity (Fig. 3g). Moreover, the CNN model is augmented with the random crop technique meaning that it can make predictions of the cropped drop images as long as the baseline, i.e., the contact between the drop and solid, is fully visible to the camera view. As a result, as shown in the new Fig. S8, the model can analyze drops that are not fully captured by the microscopy camera if the baseline, which is marked with a green line, is entirely visible.

It should be noted that the implemented ResNet 50 architecture demands a high number of training datasets if the images are fairly complex. In machine learning, this can refer to Kolmogorov complexity denoting the length of the shortest computer program that produces the image as output^54^. Therefore, in this study, the *natural* images (shown in the right column of Fig. 1b) have higher complexities as they have nonuniform grayscale colors and hues within the drops. However, *binarized* (i.e., only black and white) images are stored in few bytes, i.e., orders of magnitude less than the natural images, hence they contain a fewer number of variables that can be better correlated with the simpler geometry of binarized drops. As a result, the dimensionality reduction of the natural images through binarization would make it easier for the network to deal with simpler images and subsequently help the model converge faster if trained by a smaller number of images^55–57^. Also, considering Fig. 3j, binarizing the natural images would reduce the training time from 67 to 4 min if performed by NVIDIA Tesla T4 GPU. This explains why in Fig. 3j, the CNN model provides more accurate, stable, and rapid estimates if trained on binarized images (without using transfer learning) when compared to natural images. Besides, Fig. 3i shows that at least 200–250 unique images (shown in solid black lines) are required to sufficiently train the model and the 9 variations in the lighting condition (shown in red dashed lines) would further enhance the accuracy of the model. In other words, increasing the number of training images from 200 (200 × 9 = 1800, i.e., incorporating light variations) to 375 (375 × 9 = 3375, i.e., incorporating light variations), would not significantly increase the model accuracy, which remains at ~ 0.87 and ~ 0.93 for hydrophilic and hydrophobic test sets, respectively. It is worth noting that an accuracy measure of ~ 90% on the test set is not only realistic and ideal but conforms well to industry standards. Consequently, this figure ensures the user that the model is fully trained. Moreover, Table S1 details more optimization parameters, including the accuracy and loss values, for both the train and test datasets, in which the loss function is defined as the mean squared error between the estimated and true angles.

### Accuracy of the developed CNN algorithm

**Figure 4:**
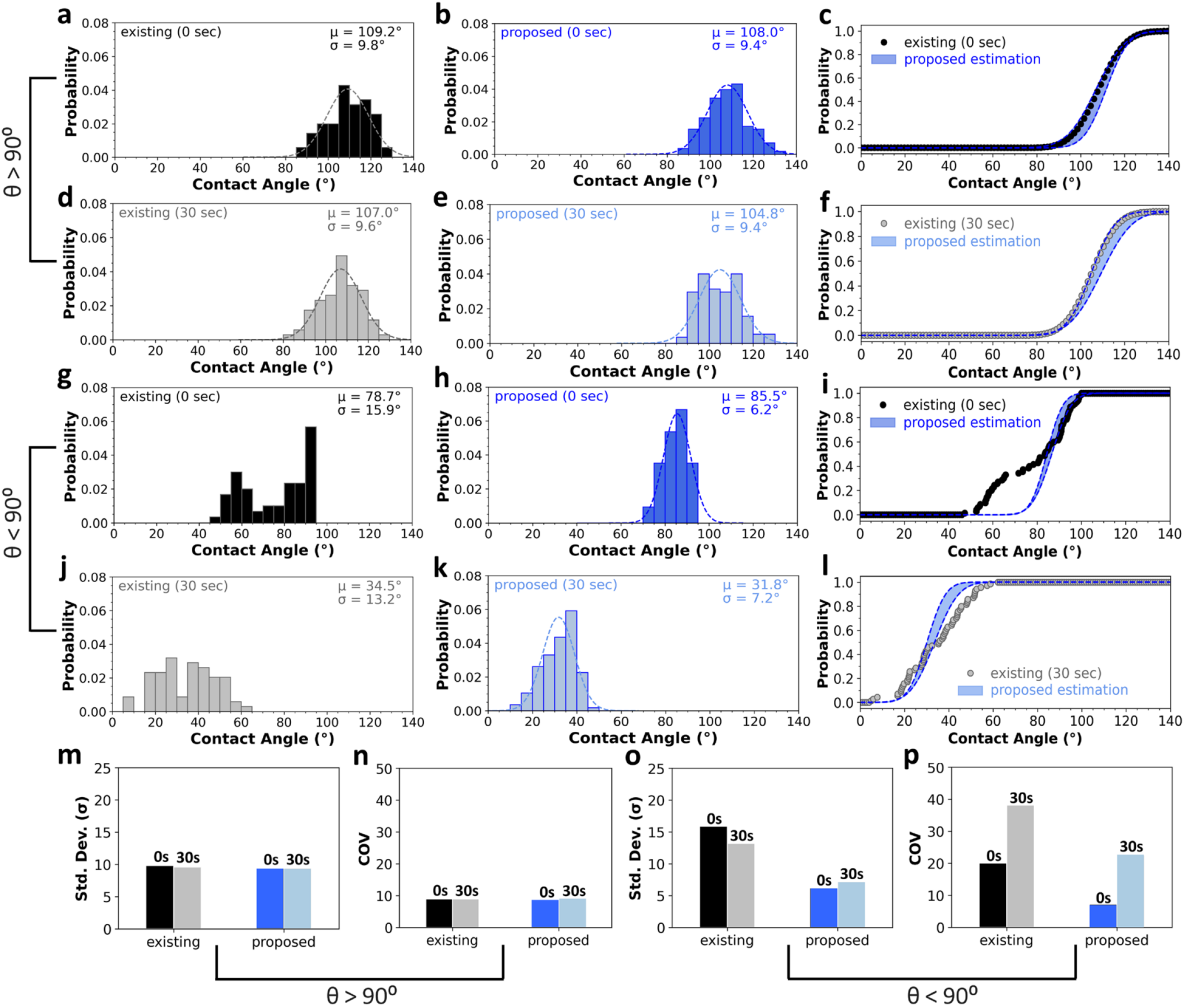
CA estimations by the existing and proposed approaches: surface wettability estimates on (**a–f**) hydrophobic (θ > 90°), and (g to l) hydrophilic (θ < 90°) surfaces at 0 and 30 s from solid–liquid interaction, and their corresponding statistical parameters including (m, o) standard deviation (σ), and (n, p) coefficient of variation (COV = µ/σ $\times$ 100). Specifically, in column 1 (**a,d,g,j**) the fitting method (existing approach), and in column 2 (**b,e,h,k**), the CNN model (proposed approach) is used to evaluate the CAs. Furthermore, column 3 (**c,f,i,l**) performs a comparison of CAs estimated by the two approaches (on each row) using the Bayesian statistics. Analysis of surface wettability on hydrophilic surfaces (third and fourth subplots of the third column) reveals a disagreement between the two approaches. Also, the COV of the CAs measured by the existing approach (at 30 s) exceeds 30% and consequently is not considered accurate, while the proposed approach presents more accurate estimates on hydrophilic surfaces.

To determine the accuracy of the developed setup, comparisons are made between the CNN-based setup (proposed approach) and the traditional goniometer (existing approach) for wettability measurements on hydrophobic and hydrophilic surfaces at 0 and 30 s from the onset of solid–liquid interaction. Considering Fig. 4, it is determined that the mean CA measurements estimated by both algorithms are almost similar; however, statistically speaking, the mean value is not a sufficient parameter to determine the accuracy of measurements. Consequently, a comprehensive Bayesian statistical analysis is leveraged to compare the accuracy of both goniometers. Considering the normality of the CNN-based CA measurements, i.e., the second column of Fig. 4 (Fig.4b,e,h,k), it is *believed* that the results of the proposed approach, called *Vague Prior*, match the CA measurements estimated by the traditional approach, named *Posterior*^58^. However, this assumption, i.e., the statistical matching of both approaches, needs to be established and verified. Accordingly, the Bayesian statistics are leveraged to calculate posterior interval estimators, i.e., mean, and standard deviation, assuming that the prior values are normally distributed. A detailed explanation of how to estimate the interval estimators is detailed in the "Methods" of this paper.

Calculating the posterior interval estimators, the corresponding PDF of the posterior values, and subsequently, the CDF envelope (shaded in blue), are estimated at different tangent angles for a 95% confidence level, see the third column of Fig. 4 (Fig. 4c,f,i,l). As shown in Fig. 4c,f, for hydrophobic surfaces, the CAs estimated by the existing approach are placed inside the blue shaded CDF envelope predicted by the Bayesian analysis, denoting that both methods can match with a 95% confidence level. However, for hydrophilic surfaces, the CAs estimated by the existing approach did not place within the corresponding CDF envelope (Fig. 4i,l). Therefore, it is required to specify which method is more accurate for surface wettability measurements. For this purpose, Fig. 4m,o compare the standard deviation (σ) of CAs estimated by the existing and proposed approaches at 0 and 30 s on hydrophobic and hydrophilic surfaces, respectively. Specifically, for hydrophobic surfaces, the average standard deviation of both approaches is 9.7° and 9.4°, respectively. However, for hydrophilic surfaces, these values change to 14.6° and 6.7°, respectively, suggesting that the results are almost twice more spread out if estimated by the existing approach.

It is worth noting that although all the tested specimens are similar, the surfaces are heterogeneous and the average standard variation of > 9° is expected if comparisons are made between 35 similar but heterogeneous surfaces (Fig. S9a). However, if 35 measurements are done on a single heterogeneous surface, the standard deviation is reduced to ~ 6° (see Fig. S9b). Furthermore, if a single metallic homogeneous surface is analyzed 35 times, the standard deviation is even smaller, i.e., ~ 3°, see Fig. S9c. As a result, the more homogeneous the surface, the smaller the variations in CA measurements will be. As previously discussed, the R-squared value between the ImageJ and CNN measurements is less than unity (Fig. 3f,g) denoting that the CNN estimations are not 100% accurate. Therefore, it is expected to have a ~ 3° standard variation based on 35 measurements on a single homogeneous specimen. Figure 4n,p show the coefficient of variation (COV) of the existing and proposed approaches. Generally, the performance of any approach is considered unacceptable if the COV of the dataset exceeds 30%^59, 60^. Considering Fig. 4, it is found that on hydrophobic surfaces, the average COV of the CAs estimated by the existing and proposed approaches are largely similar, that is, 9% and 8.9%, respectively. However, for hydrophilic surfaces, these values are increased to 14.9% and 29.2%. Specifically, on hydrophilic surfaces, the COV of the CAs estimated by the existing approach is significantly higher (38.2%) at 30 s, exceeding the 30% limit compared to 22.6% at 30 s by the proposed approach (Fig. 4p).

In the next step, for the existing goniometer, it is of interest to determine whether the error stems from hardware or software limitations. For this purpose, the image data captured by the existing goniometer is analyzed by either the existing (fitting) or the proposed (CNN) algorithms, and cross-comparisons are reported in Fig. S10. A comparison of Fig. 4 versus Fig. S10 confirms the higher accuracy of the proposed over the existing algorithm for a more accurate surface wettability assessment. Thus, the significant improvement observed in the accuracy of CA measurements via our proposed system is primarily stemming from using better software as opposed to the hardware, where the CNN outperforms the fitting goniometer.

**Figure 5:**
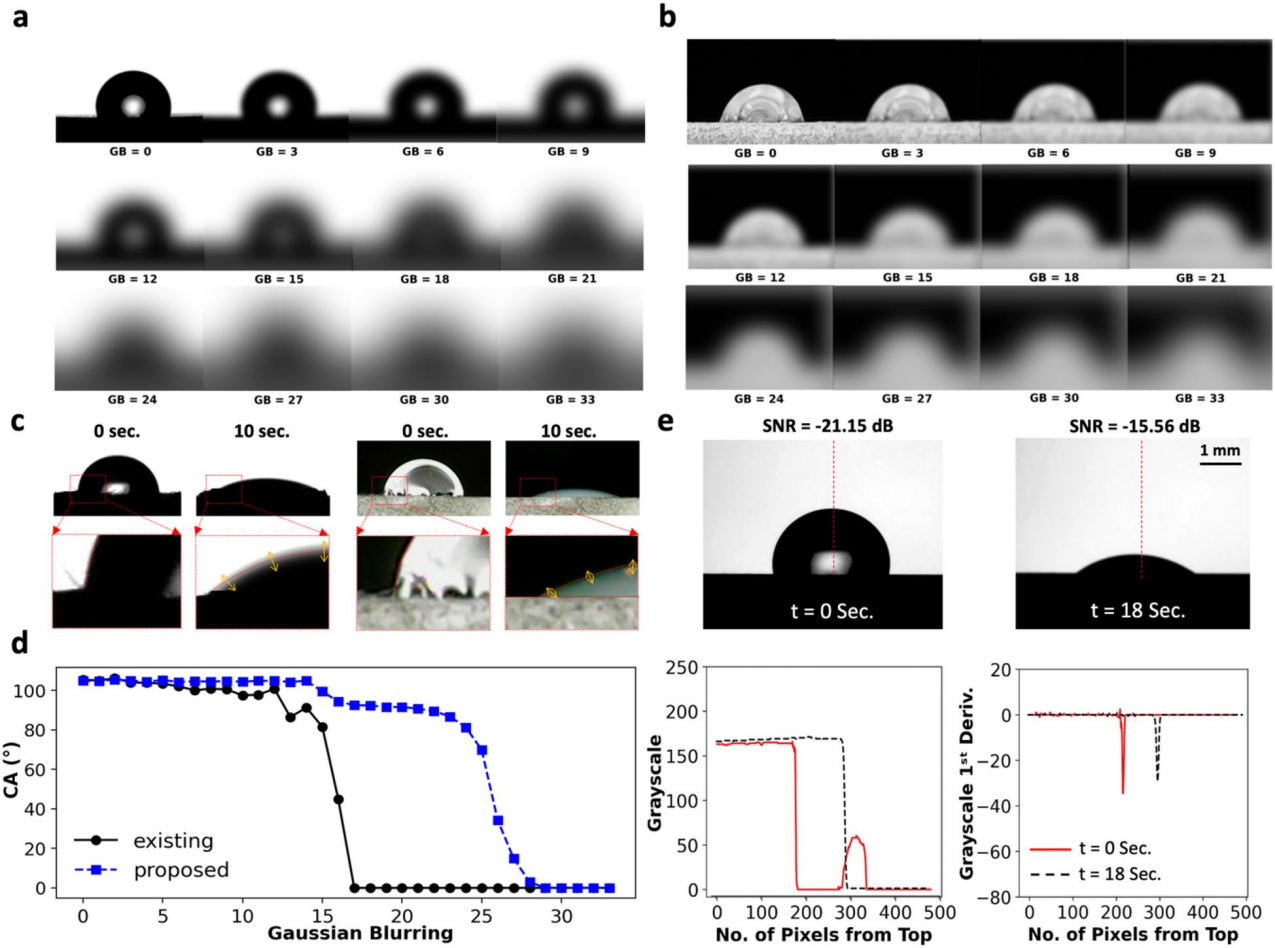
Impact of camera focus on the robustness of CA measurements: pinpointing the performance of the existing (fitting) and proposed (CNN) algorithms for surface wettability measurements of (**a**) back-lit and (**b**) front-lit images captured by the existing goniometer, and synthetically convoluted at different GB values. (**c**) Recognizing the boundary of front- and back-lit drops placed on a porous hydrophilic surface at 0 and 10 s from solid–liquid interaction using the fitting method. The ternary phase contact line is magnified, and the fitted (red) line is sketched by the algorithm along the edge of the drop. (**d**) Estimating the CAs as a function of the GB values. If the GB values exceed 12, the existing method becomes unstable and could not be used for reliable CA measurements. However, the proposed CNN model remains stable for precise analysis of more distorted images, i.e., GBs ≤ 22. (**e**) Calculating the SNR based on the variations in the grayscale values of the pixels located on the red dashed lines, proving that the drop image becomes unfocused with time.

To pinpoint the source of systematic error for the fitting algorithm, Fig. 5c shows the image data of front- and back-lit drops on a porous hydrophilic surface analyzed at 0 and 10 s. Considering this image, it is realized that at 0 s, the image is perfectly focused, and the boundary of the drop (marked with a red line) is correctly established by the fitting algorithm. However, after 10 s, due to the rapid movement of the drop on the porous hydrophilic surface, the focal length of the camera is slightly changed, resulting in an out-of-focus image. Therefore, for unfocused drop images, the fitted polynomial could not be correctly established to estimate the CAs at the ternary phase contact line. In particular, the orange arrows in Fig. 5c show uncertain regions for edge detection due to the significantly low SNR. Similarly, Fig. 5e and Video 3 estimate the SNR of drop images based on the variations in the pixel grayscale values along the red dashed lines proving that the boundary of the drop becomes unfocused from 0 (SNR = −21.15 dB) to 18 s (SNR = −15.56 dB). This means that although the camera focus is initially adjusted on both sides of the drop, the moving drop on porous hydrophilic surfaces results in an unfocused image, which is a generic issue and cannot be accurately resolved with traditional fitting algorithms. Subsequently, in the next section, we compare the stability of both techniques against the variations in the camera focus to decide which approach is more robust for wettability assessments.

### Robustness of the developed CNN algorithm

Assessing the tangent angles on the surfaces of varying wettability needs to be done by a robust CA goniometer that is less sensitive to optical limitations^42^. In fact, on *hydrophilic* surfaces, the variation in the geometry of drops is so dramatic that the optical distance of the camera changes with time, affecting the camera focus. Thus, it is necessary to establish the impact of camera focus on the reliability of measurements. Accordingly, Fig. 5a,b,d evaluates the changes in the CA of synthetically blurred images estimated by both the existing and proposed approaches. The Gaussian Blurring (GB) is a library in OpenCV that optically convolutes the image data with a low-pass filter kernel, GB = 0 denotes an unconvoluted image, while the edges of an image are softened by increasing the GB^61^. Considering Fig. 5a,b,d, it is found that the stability of the existing (fitting) algorithm is significantly affected by the camera focus, specifically for GBs exceeding 12. In contrast, the CAs estimated by the proposed (CNN) algorithm are not subjected to dramatic change as long as the GB is limited to 22, denoting the higher stability of the proposed algorithm for the analysis of optically distorted images.

It is worth noting that based on Fig. 5d if GB > 30 the CA reaches 0° which is dictated by how the ground truth is defined by the user. Basically, in Fig. S11a, the absorption of a front-lit drop on a porous hydrophilic surface is analyzed every 10 s and the corresponding average grayscale value across the image width is shown in the middle column. Next, the 1st derivative of the middle plot (shown in blue) is calculated and shown in the rightmost column of this subplot (shown in green). Finally, the SNR of the 1st derivative of the grayscale values is calculated at each time period. In particular, Fig. S11a shows that the SNR values of the absorbing front-lit drops are reduced from −17.74 (dB) to −12.19 (dB) within 40 s from the onset of solid–liquid interactions. As a result, the drop is not detectable by the camera at 40 s and the corresponding SNR value exceeds −13 dB. It is worth noting that in this step (where the drop is not detectable or fully absorbed by the porous surface), the CA is manually set to 0° as a ground truth. As a result, for back-lit (Fig. S11b) or front-lit (Fig. S11c) drops, if a drop image (captured at an arbitrary time period) is subjected to a Gaussian Blurring filter with a size of 30 or more, the corresponding SNR values would exceed −13 dB meaning that the algorithm likely assigns a CA of 0° to these images. In summary, the proposed algorithm is less sensitive to the edges of the drops and the results are mainly controlled by the overall geometry of the ROI. This means that the wettability measurements estimated by the proposed algorithm are more reliable, reproducible, and robust than the existing approach.

### Simultaneous CA measurements at different angles

One of the main advantages of the proposed goniometer is its ability to simultaneously determine the tangent angles of a drop at various angles. In fact, the measured CAs may differ from point to point along the ternary phase contact line due to the variations in the local surface heterogeneity. Therefore, the present setup increases the number of wettability measurements for a more reliable analysis of the drop compared to the traditional goniometers, which are typically equipped with a single camera. Considering Fig. S12, and Video 4, it is noticed that the mean difference between the left and right CA estimated by each camera (shaded in green) is much smaller than the mean difference between the combined average CA of the individual cameras (Fig. S12e). This explains why a single side-view of ROI is not sufficient to reliably estimate the wettability of non-spherical drops on heterogeneous surfaces. Moreover, as shown in Fig. S13 (which is a snapshot of Video 4), the maximum difference in CA estimated by the two methods, i.e., CNN and ImageJ, is never more than ~ 5°.

## Discussion

This study introduces an autonomous, robust, low-cost, and accurate CA goniometer that works under a variety of front-lit illumination conditions, liquids of various compositions and colors, and on surfaces of varying reflectivity. Specifically, an original dual-camera goniometry is coupled with a robust CNN algorithm that (unlike traditional fitting algorithms) can accurately predict the CA of even relatively flat drops on chemically homogenous solid surfaces, see Video 1. Basically, as shown in Fig. S5, experienced users (who can work with Image J) can properly create the ground truth dataset for training the CNN algorithm so that it can provide unbiased estimates of surface wettability. Furthermore, the employed algorithm is written with Pytorch, i.e., an open-source Python package, which makes it freely accessible and distributable, while traditional goniometers only work with included licensed software packages. Considering traditional goniometers, they typically leverage Contour fitting algorithms to calculate the tangent line at the solid–liquid interface. However, uncertainty as small as 1 µm in the baseline location triggered by optical distortion, e.g., camera focus and resolution, can potentially change the CA significantly, resulting in a ± 10° error^62^.

In particular, on hydrophilic surfaces, our setup provides more accurate measurements compared to the existing fitting algorithm. To justify this phenomenon, it is theorized that the rapid movement of water drops on hydrophilic surfaces has the potential to dramatically change the optical distance of drops from the camera lens and affect the curve-fitting process. To remove these inherent systematic errors from measurements, the employed CNN estimates surface wettability based on the uniqueness and overall geometry of each front-lit drop imaged in a dark room. Subsequently, the proposed setup provides more stable measurements and remains insensitive to the camera focus for a wider optical distortion range (0 ≤ GB ≤ 22) compared to the existing technique (0 ≤ GB ≤ 12)*.*

As a result, the ability to accurately analyze unfocused drop images obviates the need for subjective camera calibrations, but more importantly, it enables autonomous CA measurements using low-cost and low-resolution cameras. Although the commercially available semi- or fully automated CA goniometers have been reported to dramatically improve the speed of analysis, they are dependent on the measurement setup, and often fail to provide reliable surface wettability measurements under non-standard conditions due to three main reasons: (1) presence of non-optimal or nonuniform lighting conditions^63^, (2) differences in contrast between the drop and solid surface^3^, and (3) improper selection of contrasting background color^64^. Therefore, designing a fully automated model that is insensitive to the lighting condition is considered a huge improvement over traditional goniometers. In addition, the present goniometer characterizes surface wettability regardless of the skill and experience of the user. Finally, Table S2 summarizes the practical use efficiency of the proposed setup over the existing goniometers in terms of camera calibration, acutance requirement, software requirement, objectivity of measurements, sensitivity to light intensity, microscopy assembly, imaging condition, and capital cost. Consequently, the present straightforward, accurate, inexpensive, robust, and fully automated procedure has the potential to be widely accepted and used for accurate surface characterization with proper adaptation to any wetting system. However, our proposed setup is not currently designed/ trained to measure surface tension, interfacial tension, or surface dilatational elasticity which will be explored in the future.

## Methods

### Lab orthogonal camera goniometer setup and operation

The developed setup for CA measurement comprises a leveled and adjustable XYZ mechanical stage with two orthogonally aligned digital microscope cameras. The cameras have approximately 10 µm spatial resolution and are equipped with eight built-in LED light sources to capture the front-lit images. The height of the cameras is adjustable such that the actual drop and its reflection could be clearly captured in both directions, the 3D schematic of the setup is shown in Fig. 1a. Basically, as opposed to traditional goniometers, where the drops are analyzed using a single view, the developed setup characterizes surface wettability via two independent viewpoints.

### Traditional goniometer

Surface wettability measurements are partly done by the Ramé-Hart 250-F1 Contact Angle equipped with a leveled mechanical base, and a single external light source. The camera used in this goniometer has also 10 µm spatial resolution and uses commercial DROPiA software to recognize the boundaries of drops, which is based on a contour-based fitting algorithm. It is worth noting that for each CA measurement, the camera has to be manually focused, tuned, and calibrated on the ROI.

### Convolutional neural networks (CNN)

**Figure 6:**
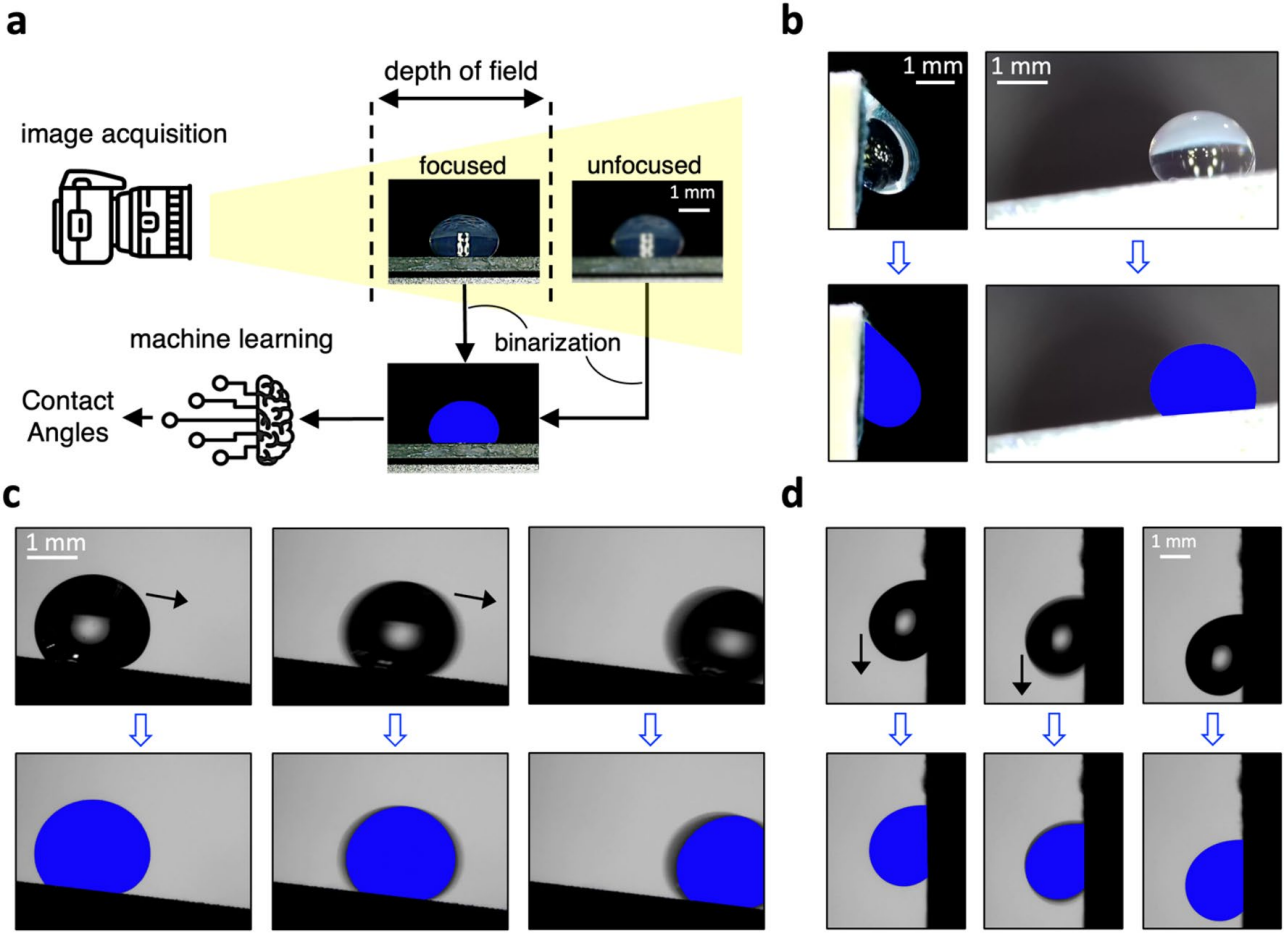
Extracting binarized masks from natural images to feed the CNN model. (**a**) Workflow that shows how the binarization algorithms obviate the need for camera auto-focusing. In the next step, the binarized maps are fed into the CNN model to create a ground truth dataset. Finally, the trained CNN model is leveraged to accurately estimate the left and right CAs of symmetrical or non-symmetrical drops based on the uniqueness and overall geometry of binarized maps. (**b**) Showcasing the ability of the binarization algorithms to extract and analyze the geometry of front-lit drops rolling down (left column) a vertical wall, and (right column) a sloped stage, (**c**) showcasing the ability of the binarization algorithm to extract and analyze the geometry of back-lit drops rolling down a sloped stage, or (**d**) a vertical wall. It should be noted that the accuracy of the proposed method is not sensitive to low SNR regions around the drop boundary triggered by capturing images via low frame-rate cameras.

As shown in Fig. 6a, the CNN model leverages the uniqueness and overall geometry of the binarized images to analyze both symmetrical and non-symmetrical drops. The CNN model is developed in PyTorch with ResNet50 architecture and its hyperparameters are fine-tuned (learning rate = 0.001, batch size = 16, and epochs = 100) to get optimum performance. Furthermore, Rectified Linear Unit (ReLU) activation function and a dropout regularization rate of 0.2 are employed to facilitate the learning of complex patterns from the input data and reduce over-fitting^65, 66^. The Max pooling layers are applied to all feature maps to decrease the dimensions of the feature maps and to reduce computational load^67^. Due to the lack of a sufficient dataset (less than 4k in total), image augmentations that include random cropping, rotation, and noise injection, are applied to the binary images to further enhance the model performance^68^. In particular, the CNN model is augmented with random rotations to improve the model prediction trained on horizontally collected binarized images. Basically, a module in torchvision named RandomRotation (degrees[, interpolation, …]) skews the ROI in either direction (clockwise or counterclockwise by up to 90°) that generalizes the model against the images captured by the camera positioned not fixed relative to the drops. Figure 6b and Video 2 show the robustness of the CNN model to analyze rolling angles placed on angled or vertical stages.

The developed CNN model is designed to analyze low-quality images (with low SNR at drop boundary) of inexpensive cameras that are not equipped with auto-focusing (see Fig. 6a) or high frame rate features, i.e., < 10 fps (see Fig. 6c,d). Also, the CNN model is trained on binary images, denoting that it retains its accuracy if the binary maps are precisely extracted from natural images, i.e., applicable to both front-lit (Fig. 6b) and back-lit drops (Fig. 6c,d). Moreover, the rightmost subplot of Fig. 6c shows the ability of the proposed model to perform predictions even if the drop is not fully visible to the camera.

### Bayesian statistics for interval estimators of mean and standard deviation

Assuming that the mean and standard deviation of the normally distributed prior values are unknown, a 100 (1 − α) % confidence level of the posterior mean $\upmu$ and standard deviation SD are calculated as^69^:

2 $$\upmu =\overline{x}\mp \frac{s}{\sqrt{n}}{t }_{n-1,{ \alpha }_{/2}},\mathrm{ SD}=[\frac{\left(\mathrm{n}-1\right){\mathrm{s}}^{2}}{{\chi }_{n-1, {\alpha }_{/2}}^{2}}, \frac{\left(\mathrm{n}-1\right){\mathrm{s}}^{2}}{{\chi }_{n-1, {1-\alpha }_{/2}}^{2}}]$$

where α is the posterior significance level estimated from the one-tailed test, $\overline{x }$ and s are the sample mean and standard deviation, respectively, and ${t}_{n-1,{ \alpha }_{/2}}$ and ${\chi }_{n-1, {\alpha }_{/2}}^{2}$ are t-student and Chi-squared distributions, respectively. Estimating the interval estimators, i.e., $\upmu$, and SD, the probability density function (PDF) and subsequently the corresponding cumulative density function (CDF) of the posterior can be calculated.

### Drop placement on solid surfaces

To estimate the CAs using the sessile drop method, the syringe needle is placed at a fixed distance of 1.5–2.5 mm from the solid surface. To eliminate the role of gravity, the diameter of drops should be smaller than the capillary length λ = √(γ/[ρ. g]), where g is gravity, γ is surface tension, and ρ is the liquid density^70^. This suggests that at room temperature (23 °C) and 50% RH, the liquid volume has to be smaller than ~ 10.7 µL. Also, if the volume is smaller than 3 µL the kinetic energy dissipation of liquid results in the rapid movement of drops, which cannot be captured by inexpensive (low frame rate) cameras^3^. Therefore, in our study, a fixed volume of 5 ± 0.2 µL sessile deionized water drop is gently placed above a vibration-free surface. As shown in the new Fig. S14, our proposed algorithm did not face any issues in recognizing the ROIs of different volumes. Moreover, this figure suggests that on horizontal surfaces (Fig. S14a) the CAs remain constant regardless of the drop volume (as long as it varies from 4 to 10 µL). However, on vertical surfaces (Fig. S14b) the advancing and receding angles change with an increase in volume. To eliminate drop evaporation from the solid surface, the duration of measurements is limited to 30 s from the onset of solid–liquid interaction. In addition, each drop is applied to a single virgin and dry surface vacuum dried for 72 h over silica gel at room temperature with zero residual water content.

## Supplementary Information

Supplementary Information.

Supplementary Video 1.

Supplementary Video 2.

Supplementary Video 3.

Supplementary Video 4.

Supplementary Legends.

## Data Availability

All data supporting the findings of the present study are available within the article and the Supplementary Information (SI). Additional data is available upon request from the corresponding author.
