# Supplementary material for: Machine learning enabled orthogonal camera goniometry for accurate and robust contact angle measurements
Source: Sci Rep. 2023 Jan 27;13:1497. doi: 10.1038/s41598-023-28763-1 (PMC9883237; doi:10.1038/s41598-023-28763-1)
Supplement: Supplementary file 1 — Supplementary Information. [file 41598_2023_28763_MOESM1_ESM.docx]

**Supporting Information for**

**Machine Learning Enabled Orthogonal Camera Goniometry for Accurate and Robust Contact Angle Measurements**

Hossein Kabir and Nishant Garg *

Department of Civil and Environmental Engineering, University of Illinois at Urbana-Champaign, Urbana, IL, United States

* Corresponding author: Nishant Garg, E-mail address: [nishantg@illinois.edu](mailto:nishantg@illinois.edu)


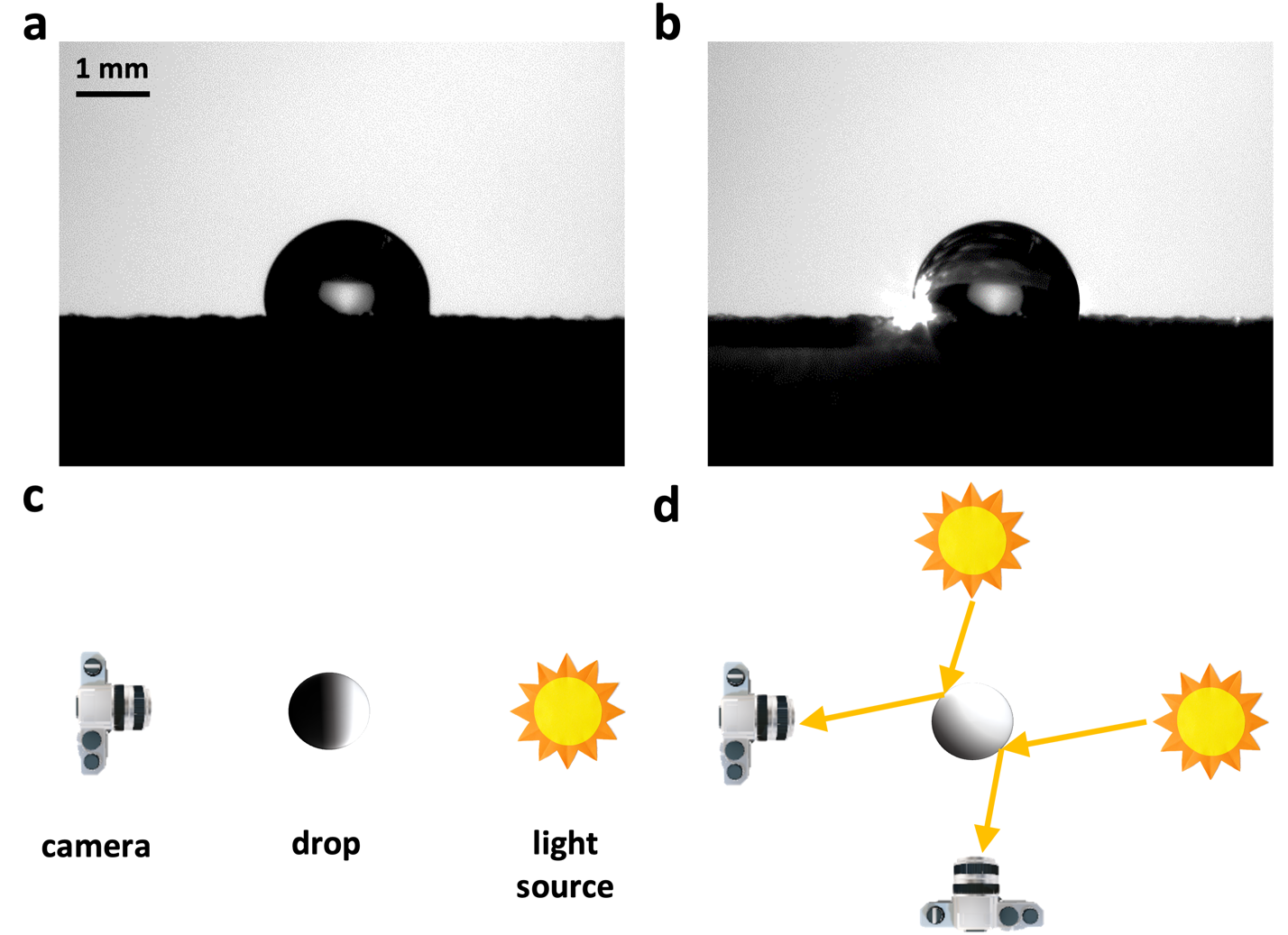


**Fig. S1: Back-lit illumination of drops for single vs orthogonally aligned cameras:** (a) back-lit illumination in a single direction, (b) back-lit illumination with orthogonally oriented illumination sources, the edge artifacts justify the need for front-lit illumination, (c) mechanism of the single illumination source, and (d) mechanisms of the orthogonally aligned illumination sources.


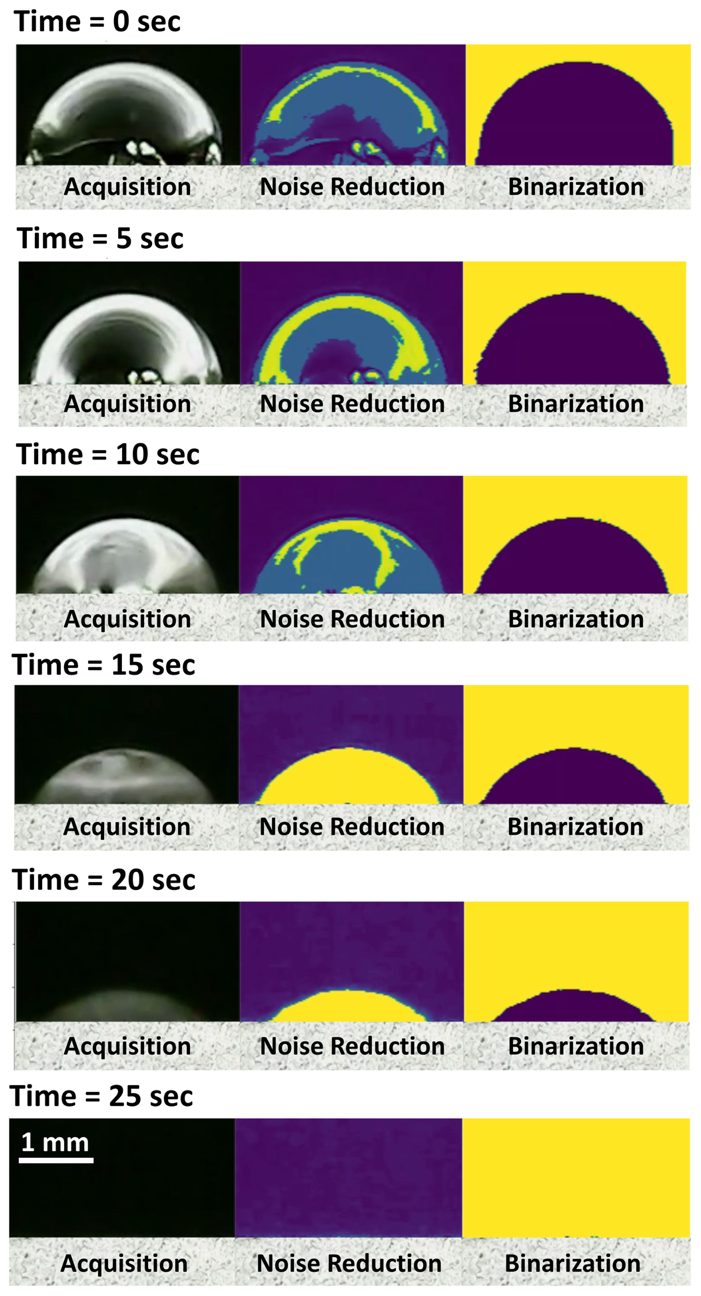


**Fig. S2**: **Noise reduction analysis of image data acquired from microscope cameras at different ages**: noise reduction filter on natural image data at 0, 5, 10, 15, 20, and 25 seconds from the onset of drop placement on a flat hydrophilic porous surface. The employed noise reduction algorithm is needed especially during the early stages (first 10 seconds) of solid-liquid interaction. This also means that no significant difference is observed between the noise-reduced and binarized images at later ages, i.e., 15 seconds and later.


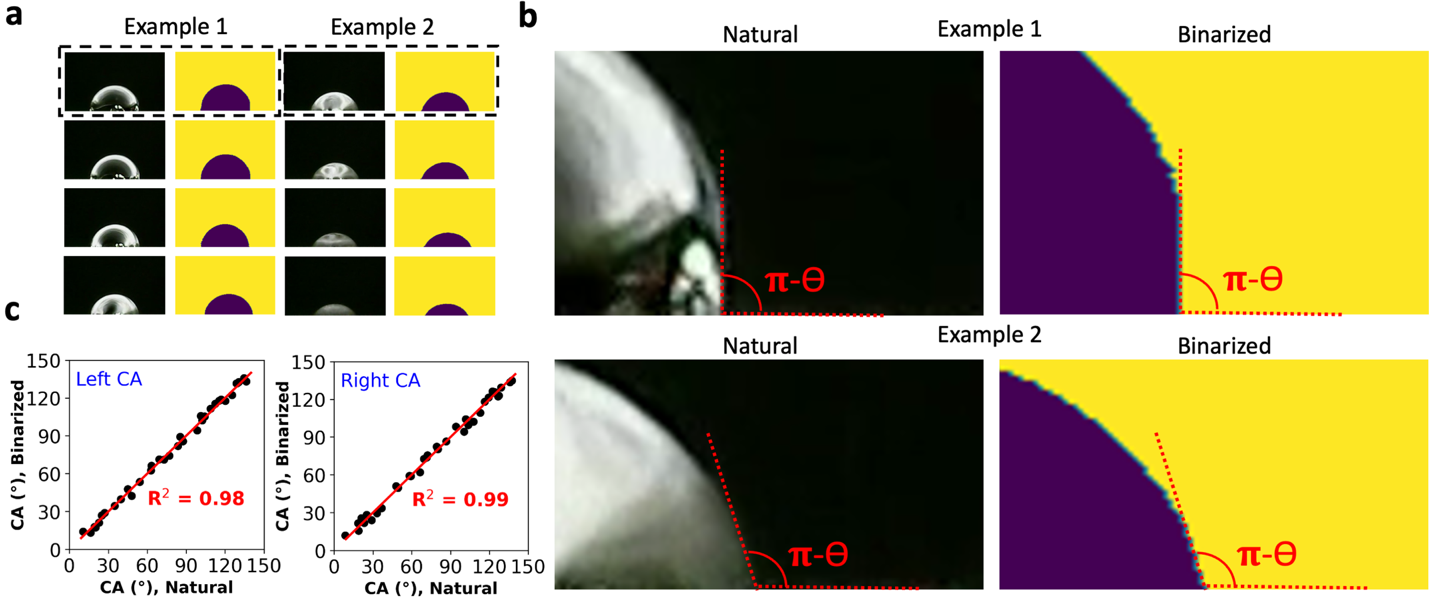


**Fig. S3**: **Effect of noise reduction and binarization on the apparent CA of drops**. (a) choosing two examples from absorbing drops on a porous substrate, (b) magnifying the ternary phase contact point of the chosen examples for natural and binarized drops, and (c) matching the left and right CAs of drops placed on surfaces of varying wettability between the natural and binarized images.


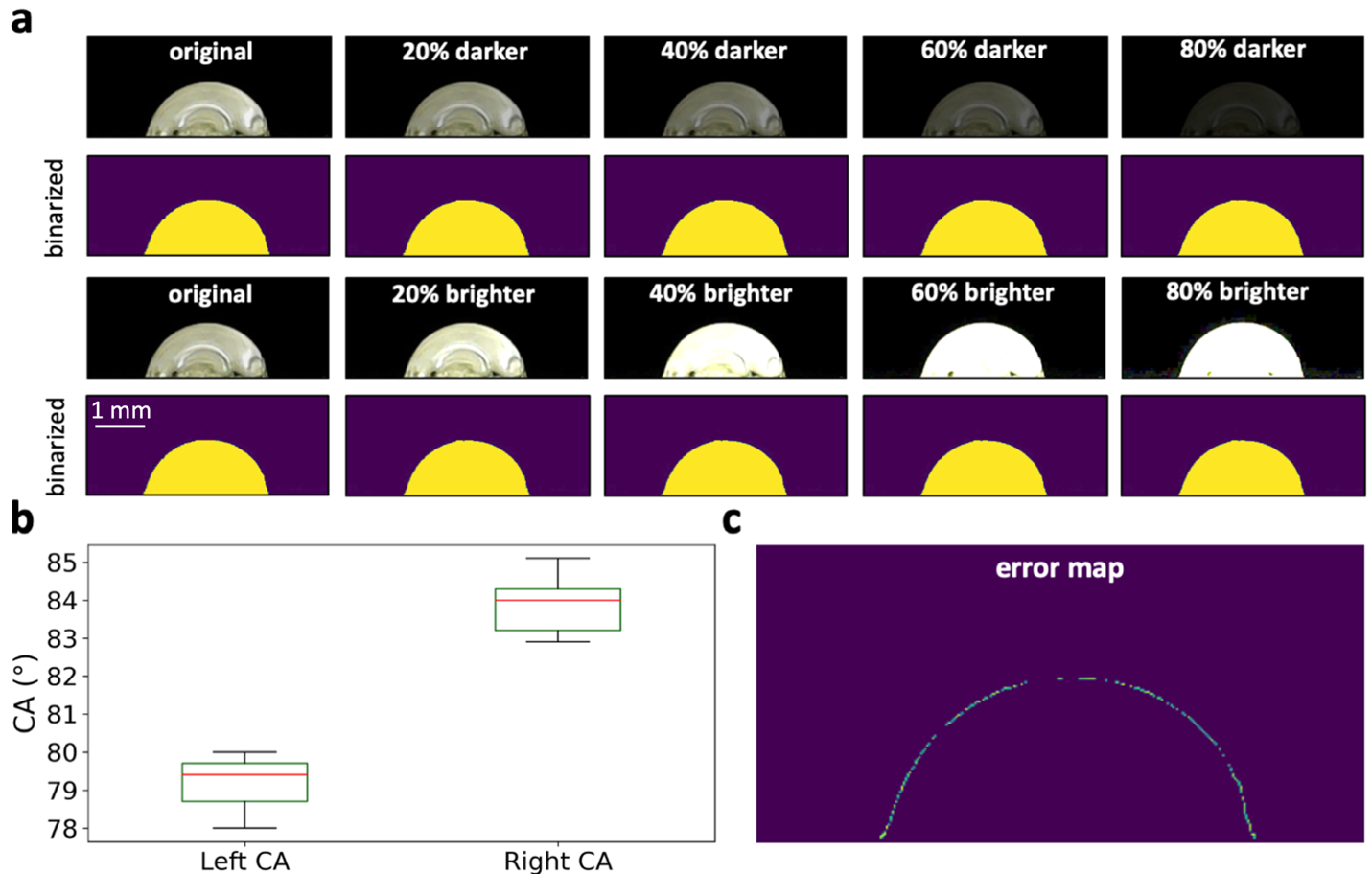


**Fig. S4: Generalizing the CNN model against the variations in the drop illumination**. (a) changing the brightness of the front-lit drop from 80% darker to 80% brighter to account for the variations in the LED lighting conditions, and estimating their corresponding binarized maps, (b) calculating the variations in the manually estimated CAs caused by the changes in the brightness, and (c) calculating the (max-min) error map between the binarized maps of a single drop at different lighting conditions.


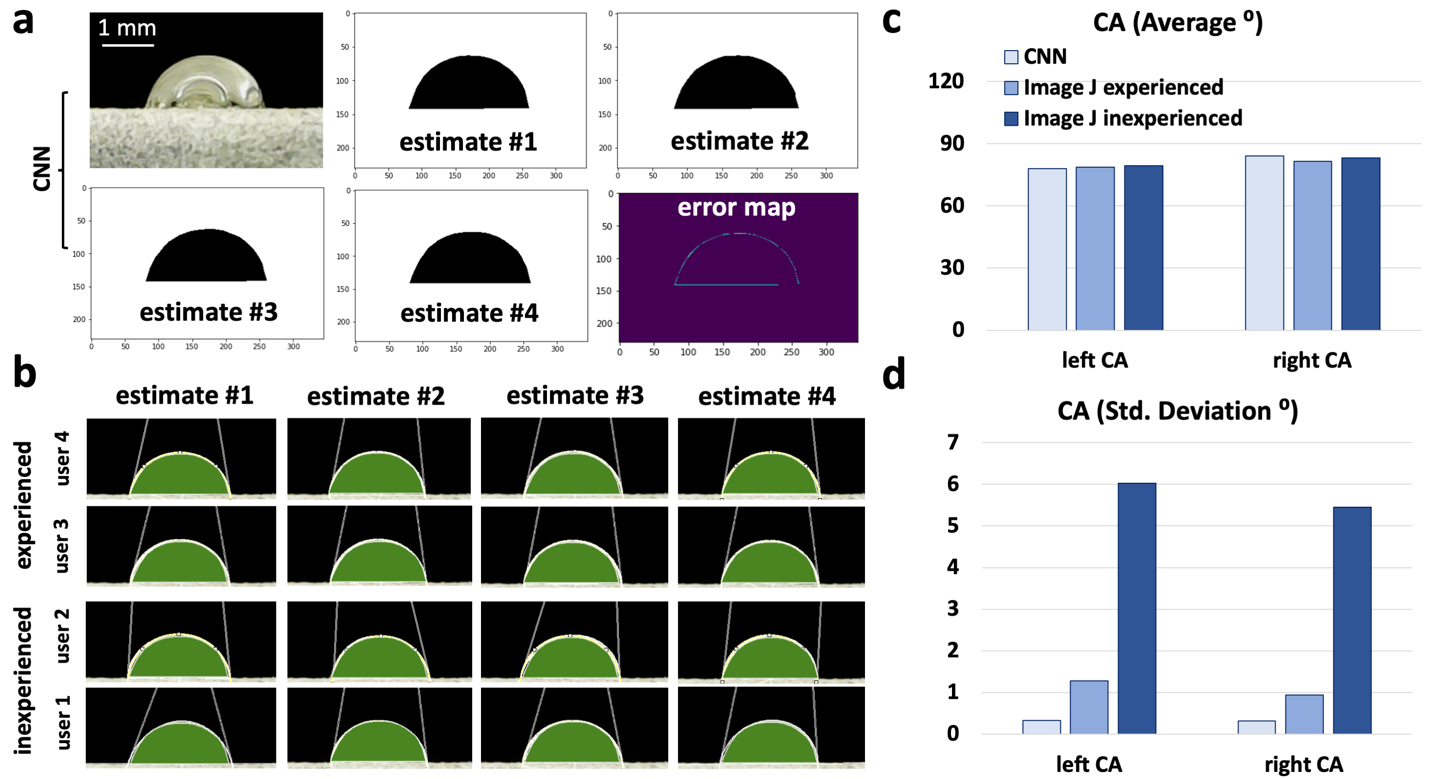


**Fig. S5**: **Comparing the performance of ImageJ vs the proposed CNN method for surface wettability assessment**: (a) ability of the developed CNN model to reproduce similar CA estimates of a single drop, (b) ImageJ- based CA measurements by experienced and inexperienced users on a single drop, (c) comparing the average and (d) standard deviation of CA measurements between the manual (human) and automated (CNN) estimates.

**Table S1**: **Optimizing metric of the employed machine** (after 100 training cycles/ epochs): the test sets have 70 separate drop measurements on hydrophobic or hydrophilic surfaces.

| Parameters | Train | Test (Hydrophobic) | Test (Hydrophilic) |
| --- | --- | --- | --- |
| Loss | 0.0472 | 0.0725 | 0.0984 |
| Accuracy (R^2^) | 0.9951 | 0.9289 | 0.8734 |


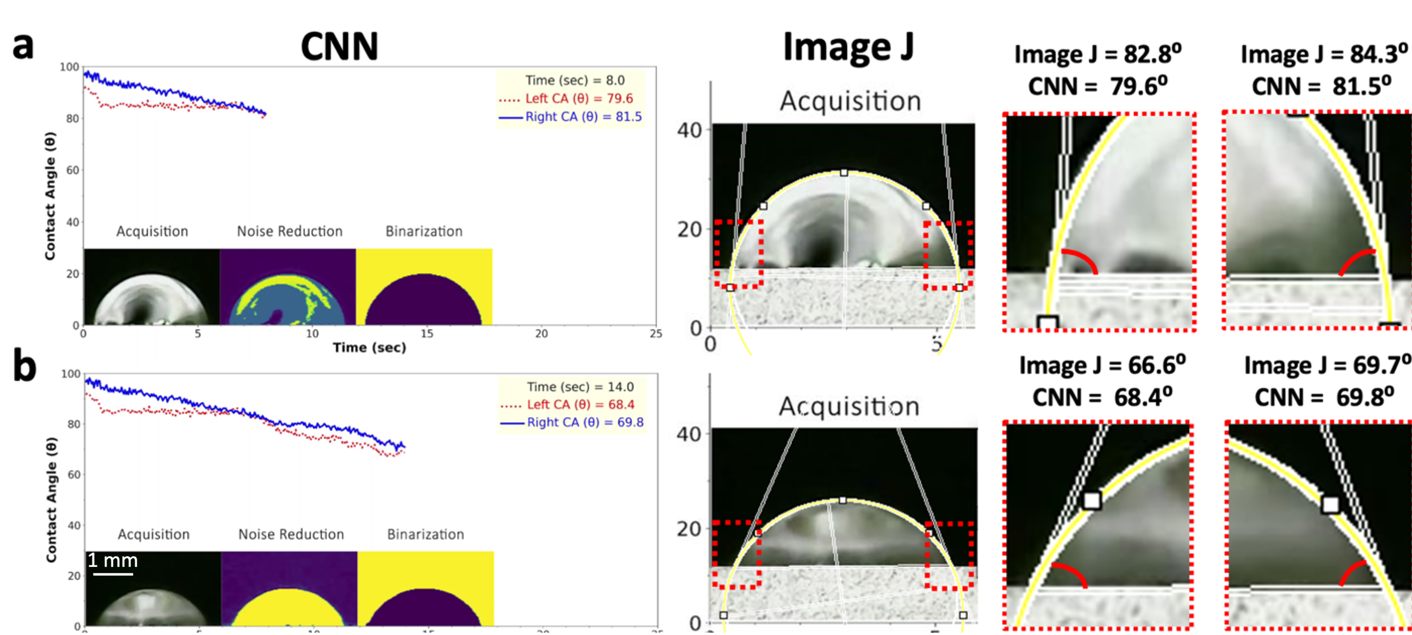


**Fig. S6**: **Comparing the CA measurements made by the proposed CNN and ImageJ for a drop placed on a porous hydrophilic surface:** at (a) 8 seconds, and (b) 14 seconds from the beginning of solid-liquid interaction.

**
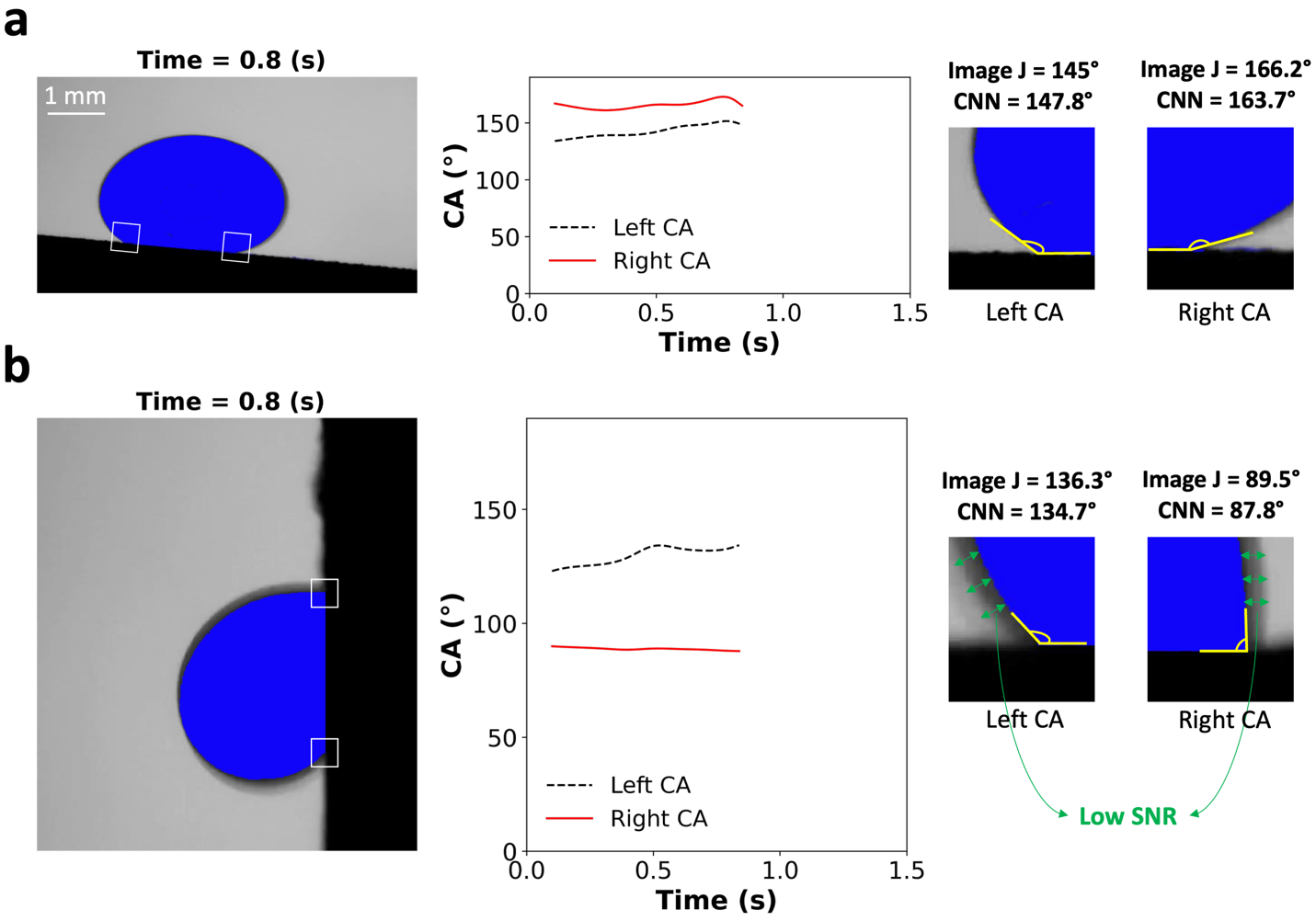
**

**Fig. S7**: **Comparing the CA measurements made by the proposed CNN and ImageJ for non-symmetrical drops:** on (a) vertical wall, and (b) angled stages at 0.8 seconds from the onset of solid-liquid interaction. Note that the method retains its accuracy even if the SNR is relatively low at the drop boundary.


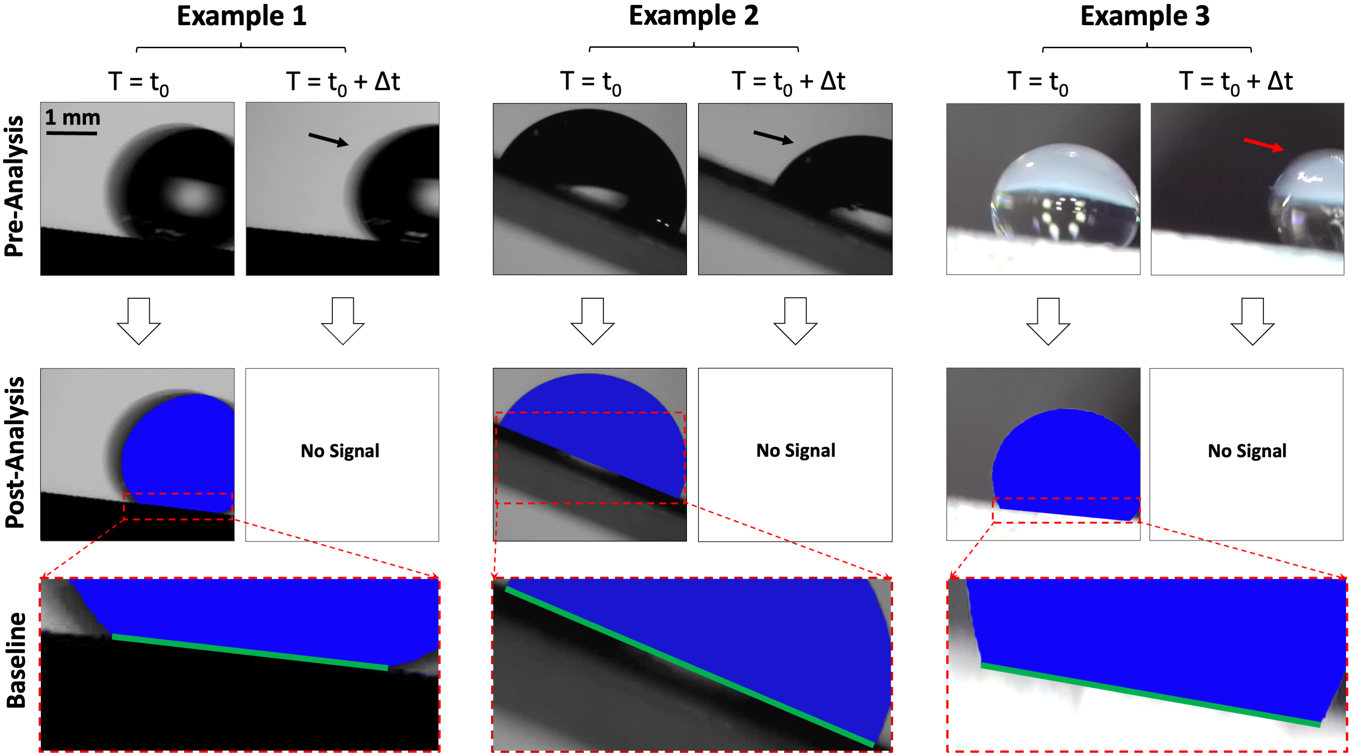


**Fig. S8**: **Ability of the CNN model to analyze cropped drop images:** This figure proves the ability of the model to analyze the drop images that are not fully visible to the camera as long as the baseline (marked with a green line in the last row) is entirely observable.


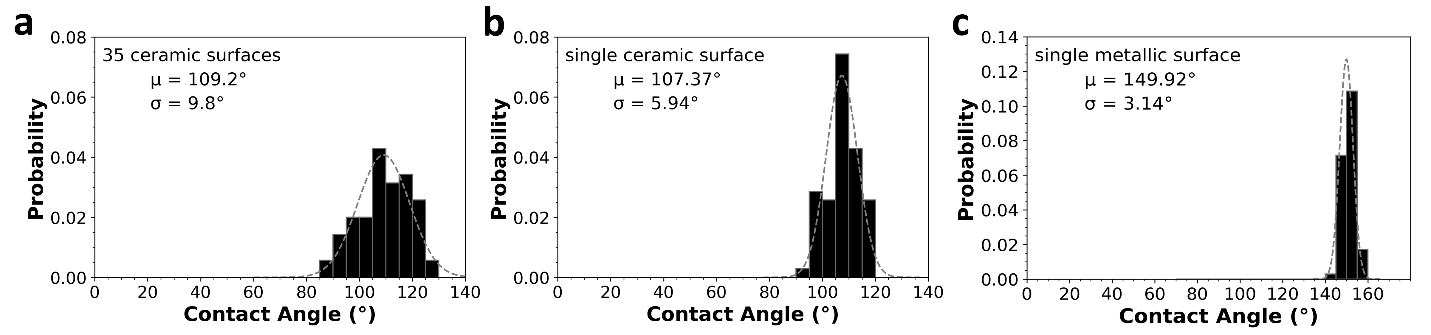


**Fig. S9**: **Variability of surface wettability measurements** **on different surfaces:** (a) 35 similar but heterogenous ceramic surfaces, (b) 35 measurements on a single heterogeneous ceramic surface, and (c) 35 measurements on a single homogeneous metallic surface.


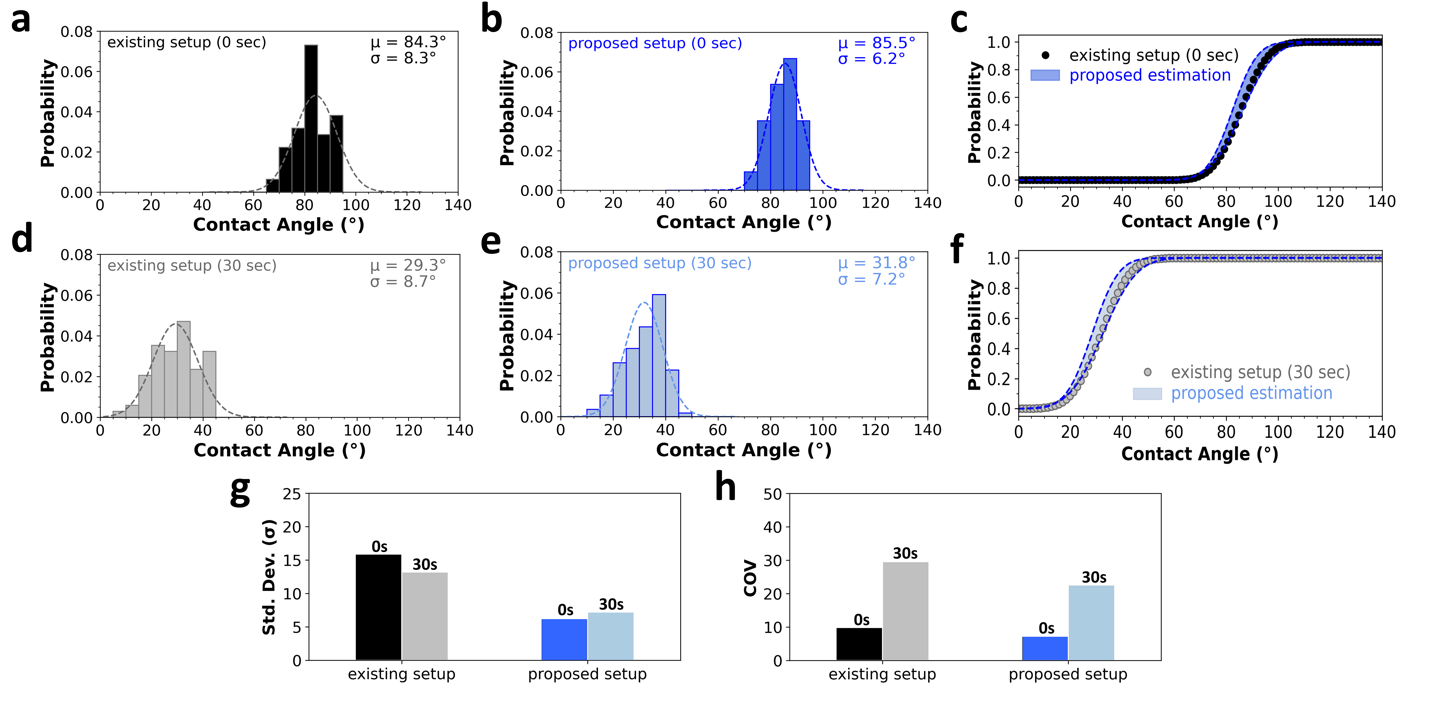


**Fig. S10: CA estimations made by the developed CNN coupled with the existing and proposed goniometers:** the CNN-based CA estimation on hydrophilic (θ < 90°) surfaces at (a-c) 0 seconds, and (d-f) 30 seconds from the onset of solid-liquid interaction, and their corresponding statistical parameters including (g) standard deviation (σ), and (h) coefficient of variation (COV). In column 1, the fitting algorithm is substituted with the CNN model to estimate the CAs of images captured by the existing commercial goniometer. In column 2, the CNN model is used to estimate CAs based on the image data captured by the proposed goniometer. Column 3 compares the CA measurements of each row using the Bayesian statistical analysis.


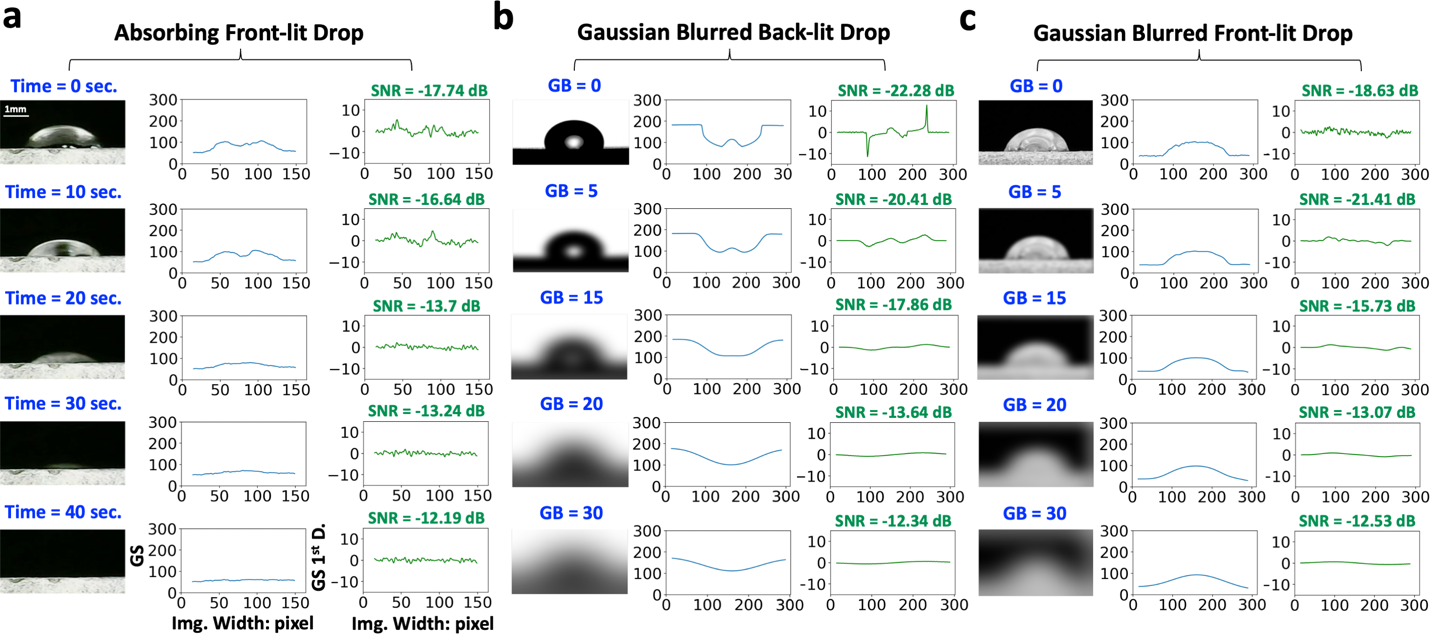


**Fig. S11**: **Role of the Gaussian Blurring filter on the stability of CA measurements.** (**a**) (left column) capturing the images of an absorbing front-lit drop placed on a porous hydrophilic surface, (middle column) measuring the average grayscale values across the image width, and (right column) finding the 1^st^ derivative of the grayscale values and estimating the corresponding SNR values in dB, doing similar procedures for (b) back-lit and (c) front-lit drops subjected to the Gaussian Blurring filters of different sizes ( 0 to 30). This figure justifies why the drop image becomes undetectable if the GB = 30 as in this condition the absolute SNR drops below 13 dB.


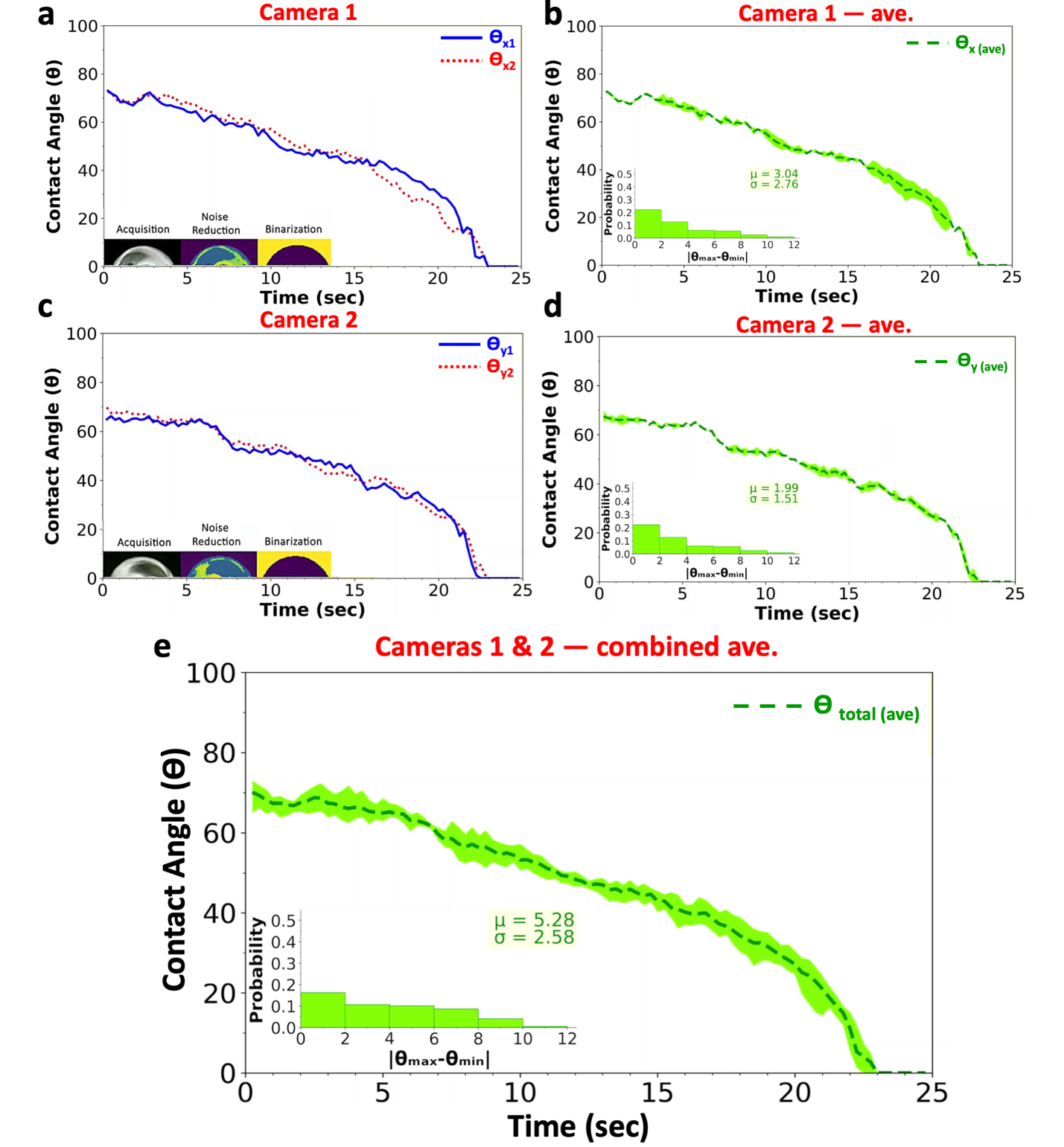


**Fig. S12: Estimating the CAs of non-spherical drops on heterogeneous hydrophilic surfaces with the proposed dual camera setup:** (a) using the C1 view, (b) average CA estimation of the C1 view, (c) using the C2 view, (d) average CA estimation of the C2 view, (e) average CA of the combined C1 and C2 views. The shaded regions (in green) show the (max-min) error.


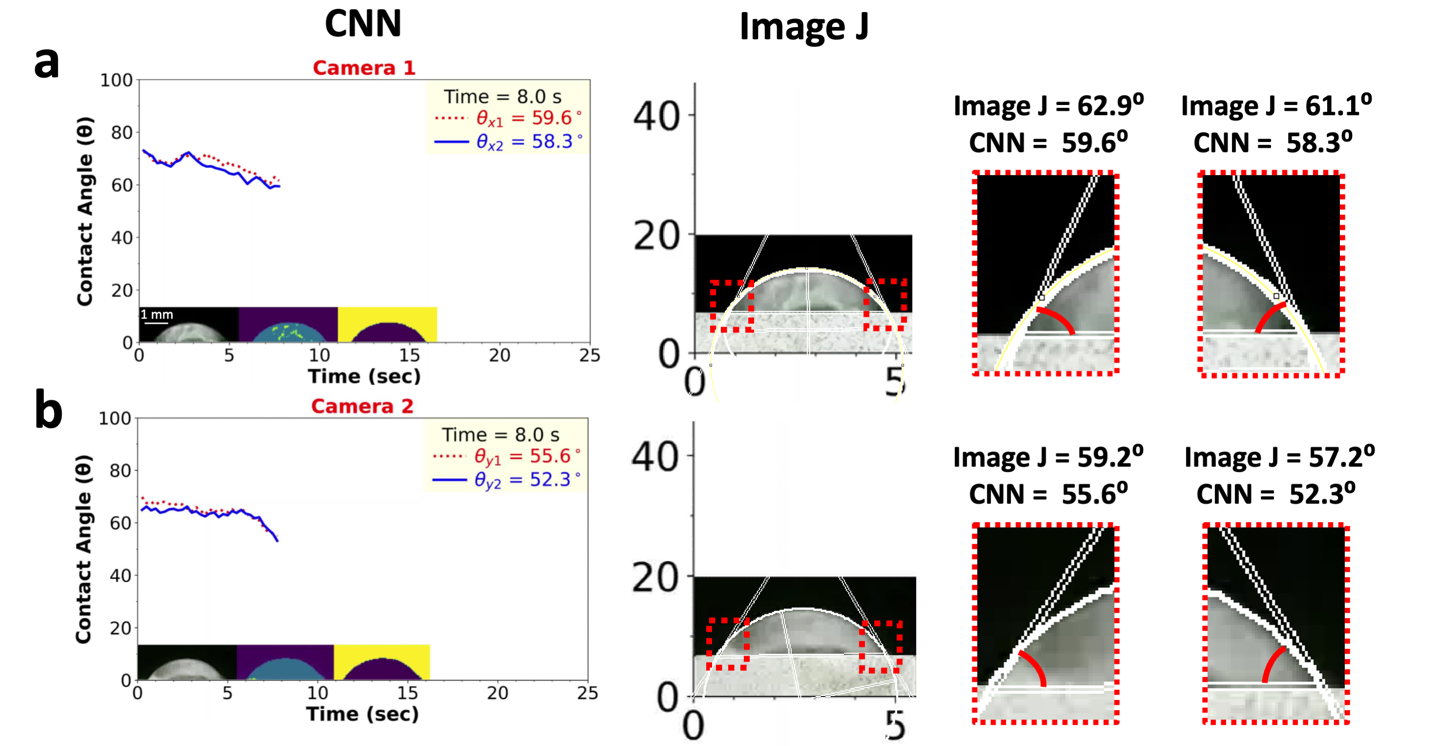


**Fig. S13**: **Comparing the CA measurements made by the proposed CNN and ImageJ for a drop placed on a porous hydrophilic surface:** at 8 seconds from the beginning of solid-liquid interaction viewed by (a) camera 1, and (b) camera 2.


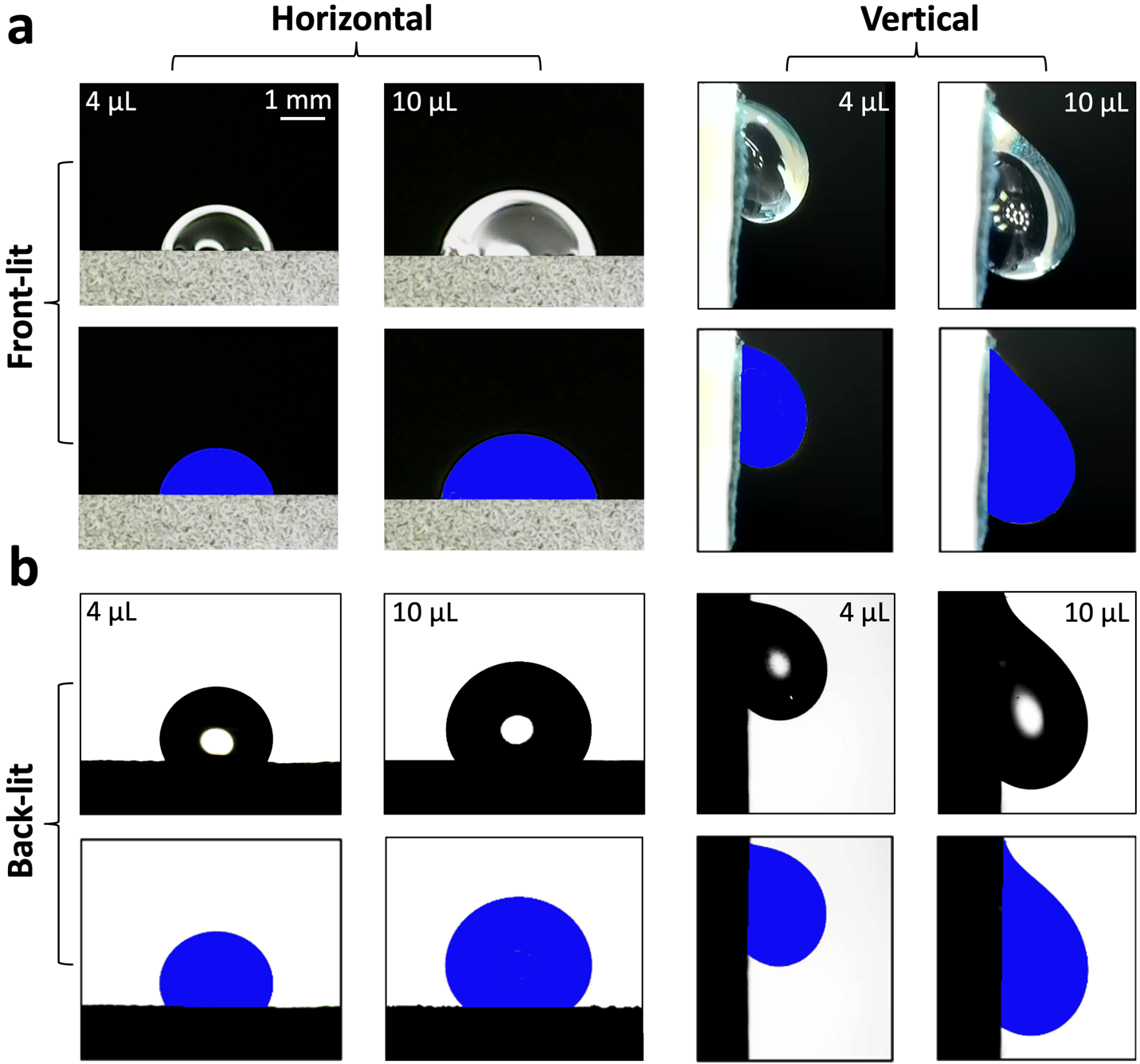


**Fig. S14**: **Impact of the drop volume and orientation on the CA values.** (a) front-lit and (b) back-lit drops placed on (left subplot) horizontal and (right subplot) vertical surfaces. On horizontal surfaces, the CAs are independent of the drop volume, while on vertical surfaces, the advancing and receding angles are subject to change with volume.

**Table S2**: Marking the practical use efficiency of the proposed setup over the existing goniometers.

| Feature | Proposed Goniometer | Existing/ Traditional Goniometers |
| --- | --- | --- |
| Need for Camera Calibration? | No, the setup retains its full accuracy until the camera remains functional. Besides, the proposed goniometer recognizes the overall geometry of the drop, hence (unlike Contour fitting method) is not sensitive to the optical noises of the image. | Yes, every month (or every time) that the traditional goniometers are moved, it is recommended to precisely re-calibrate using the exact same steps that were used for the first time. It is also suggested to check the calibration on a daily basis. |
| Acutance Requirement? | No, the proposed method can analyze even unfocused drops to delineate the boundaries with low SNR. | Yes, for sphere or rod calibrations, the user has to manually move the stage toward or away from the camera that has at least 1024 x 768 pixels resolution. |
| Open-Source Software? | Yes, the ML program is written with Pytorch (an open-source Python package) making it freely accessible and distributable. | No, the commercial goniometers only work with included licensed software packages. |
| Objective Measurements? | Yes, if a sufficient dataset is prepared by an experienced user to fully train the ML model. Moreover, the trained model can accurately analyze relatively flat (< 20$^{\circ}$) drops. | No, the measurements would be subject to significant change based on the skill and experience of the user. Also, the repeatability of measurements deteriorates for almost flat (< 20$^{\circ}$) drops. |
| Sensitivity to Back- or Front-Lit Intensity? | No, the proposed method is not sensitive to the intensity of the back-lit illumination. Besides, the algorithm is augmented with noise injection to improve the performance of the model. | Yes, if the front-light is too bright, the camera will be saturated and distort the results. Also, for low light intensity, specks, and dots, i.e., dust, will appear around the drops, which causes errors. |
| Microscopy Assembly? | Two orthogonal low-frame-rate USB microscopy cameras that cost $25 each. | A single high framerate (>100 fps) microscopy camera equipped with a 10X Ramsden-type eyepiece and a 2.3X objective lens that costs at least $4.5k. |
| Imaging Condition? | The sample (liquid drop) and mechanical stage together can cover almost 100% of the live image view given that the contact line is fully visible to the USB cameras. | The sample (i.e., liquid drop) and mechanical stage together should cover less than ~20% of the live image view. Also, the dispensing syringe should be removed from the view prior to analysis. |
| Capital Cost? | $175 (no major maintenance is required). | $35-60k (excluding major biannual maintenance/ calibration costs). |
