# Supplementary material for: Machine learning enabled orthogonal camera goniometry for accurate and robust contact angle measurements
Source: Sci Rep. 2023 Jan 27;13:1497. doi: 10.1038/s41598-023-28763-1 (PMC9883237; doi:10.1038/s41598-023-28763-1)
Supplement: Supplementary file 6 — Supplementary Legends. [file 41598_2023_28763_MOESM6_ESM.docx]

**Machine Learning Enabled Orthogonal Camera Goniometry for Accurate and Robust Contact Angle Measurements**

Hossein Kabir and Nishant Garg*

Department of Civil and Environmental Engineering, University of Illinois at Urbana-Champaign, Urbana, IL, United States

* Corresponding author: Nishant Garg, E-mail address: [nishantg@illinois.edu](mailto:nishantg@illinois.edu)

**Video 1**: CNN-based CA measurements from a horizontal viewpoint.

**Video 2**: CNN-based CA measurements from an angled viewpoint.

**Video 3**: SNR estimations based on pixel grayscale values.

**Video 4**: CNN-based CA measurement from double horizontal viewpoints.
